# Exploration of urinary metabolite dynamicity for early detection of pregnancy in water buffaloes

**DOI:** 10.1038/s41598-022-20298-1

**Published:** 2022-09-29

**Authors:** Archana Sarangi, Mayukh Ghosh, Suman Sangwan, Rajesh Kumar, Sunesh Balhara, S. K. Phulia, R. K. Sharma, Subhasish Sahu, Sandeep Kumar, A. K. Mohanty, A. K. Balhara

**Affiliations:** 1 ICAR-Central Institute for Research On Buffaloes, Hisar, 125001 India; 2 Department of LPM, LUVAS, Hisar, 125001 India; 3 ICAR-National Bureau of Plant Genetic Resources, Pusa Campus, New Delhi, 110012 India; 4 ICAR-National Dairy Research Institute, Karnal, 132001 India; 5 Present Address: Department of Veterinary Physiology and Biochemistry, RGSC, BHU, Mirzapur, Uttar Pradesh 231001 India; 6 Present Address: Department of Veterinary Physiology and Biochemistry, LUVAS, Hisar, 125001 India

**Keywords:** Biotechnology, Developmental biology, Molecular biology, Physiology, Biomarkers, Molecular medicine

## Abstract

Early and precise pregnancy diagnosis can reduce the calving interval by minimizing postpartum period. The present study explored the differential urinary metabolites between pregnant and non-pregnant Murrah buffaloes (*Bubalus bubalis*) during early gestation to identify potential pregnancy detection biomarkers. Urine samples were collected on day 0, 10, 18, 35 and 42 of gestation from the pregnant (*n* = 6) and on day 0, 10 and 18 post-insemination from the non-pregnant (*n* = 6) animals. ^1^H-NMR-based untargeted metabolomics followed by multivariate analysis initially identified twenty-four differentially expressed metabolites, among them 3-Hydroxykynurenine, Anthranilate, Tyrosine and 5-Hydroxytryptophan depicted consistent trends and matched the selection criteria of potential biomarkers. Predictive ability of these individual biomarkers through ROC curve analyses yielded AUC values of 0.6–0.8. Subsequently, a logistic regression model was constructed using the most suitable metabolite combination to improve diagnostic accuracy. The combination of Anthranilate, 3-Hydroxykynurenine, and Tyrosine yielded the best AUC value of 0.804. Aromatic amino acid biosynthesis, Tryptophan metabolism, Phenylalanine and Tyrosine metabolism were identified as potential pathway modulations during early gestation. The identified biomarkers were either precursors or products of these metabolic pathways, thus justifying their relevance. The study facilitates precise non-invassive urinary metabolite-based pen-side early pregnancy diagnostics in buffaloes, eminently before 21 days post-insemination.

## Introduction

Livestock is crucial for global food security and livelihoods. Although demand for animal products is anticipated to increase significantly in the near future, the ensuing climate change will intensify the competition for resources, further demanding enhancement in productivity as well as efficiency from animal husbandry practices. The buffaloes, contributing about half of total milk production and 18.85% to meat production, are the backbone of Indian livestock sector^1^. Moreover, buffalo are gradually preferred as livestock species for dairy farming due to their inherent advantages, such as improved feed conversion efficiency and higher economic returns in terms of higher fat, milk and meat production. Considering 2003 as the base year, an approximately 18% increase is expected in the buffalo population by the year 2023^2^. In India, the buffalo population in 2003 was reported to be 97.9 million and that has increased to 109.85 million in 2019, gaining almost 12% as per the outcome of the 20th Livestock Census by the Department of Animal Husbandry & Dairying (DAHD), Govt. of India^3^. Among all buffalo breeds, Murrah is termed as ‘black gold' in India due to high lactation yields and efficient adaptation to the dry plane topography. According to the statistics of Breed Survey 2013 by DAHD, Govt. of India, the share of Murrah was 44.39% (48.25 million) of the entire Indian buffalo population (108.70 million) in 2012^4^.

The production profile of livestock is a flipside of their reproductive performance. Early pregnancy diagnosis alongside the factors regulating the smooth progression of healthy pregnancy are important aspects for optimizing production as it can reduce the calving interval by identifying the non-pregnant animals, their timely treatment and rebreeding to optimise the postpartum interval. Early and accurate pregnancy diagnosis is pivotal to avert the economic loss exerted through undesired extension of the open period by delayed insemination in non-pregnant animals and slaughtering of the pregnant animals resulting from improper pregnancy diagnosis^5,6^. The most commonly employed pregnancy diagnosis methods in buffaloes include per-rectal palpation that can detect pregnancy accurately but not earlier than 32 to 35 days post-insemination, while the other method, transrectal ultrasonography, serves the purpose only after 25 days of insemination^7,8^. As the estrous cycle repeats after 21 days and both the aforesaid methods are unable to detect pregnancy within 21 days of post-insemination, so escape of at least one estrous cycle in case of every unsuccessful conception stands inevitable. Further, the probability of escaping the estrous cycle in buffaloes is higher than other bovines as 15–73% of buffaloes present silent heat symptoms, particularly in the summer season^5,9^. So, the necessity of an alternate pregnancy diagnostic method in buffaloes before 21 days of post-insemination is still well-perceived and vividly justifies the objective of the current study.

Nuclear magnetic resonance (NMR) spectroscopy is a non-destructive technique to elucidate the structures and dynamics of molecules present in a biofluid. In NMR, the atomic species of the analytes under a static magnetic field exhibit differential nuclear spins when a second, time-dependent magnetic field is applied perpendicular to the static one, resulting in transitions of nuclear magnetic energy levels of the atomic species, yielding typical resonance spectra corresponding to the existing atomic species^10^. NMR-based urinary metabolite profiling has been depicted as one of the most basic, yet efficient techniques to be extensively employed in biomarker discovery of diverse patho-physiological states across species^11–20^. In cattle, the most notable change in maternal metabolite profile was recorded around day 14–19 of implantation, when the process of maternal recognition of pregnancy took place with attachment of the filamentous blastocyst to the placental surface along with increased utero-placental blood flow with marked changes in the level of associated metabolites in blood and urine^21,22^. However, a promising outcome in NMR-based introspection of cervical mucous from dairy cows after 5 to 15 days of breeding has been reported to predict the pregnancy outcome with 94–98% efficiency based upon the assessment of peak asymmetry indices^23^. Maternal plasma metabolite dynamics during progression of healthy pregnancy in sheep has been explored through ^1^H NMR analysis. At four different time-points of gestation (50, 70, 90, and 110 days), thirteen significantly varying metabolites pertaining to amino acid metabolism and lipid metabolism have been identified, thus depicting the potential of this analytical modality to elucidate pregnancy-induced metabolic adjustments^24^. Serum metabolite biomarkers for early detection of pregnancy and prediction of litter size in sheep has recently been explored through liquid chromatography coupled with tandem mass spectrometry and NMR analysis. The elucidated panel of biomarkers has depicted prediction efficiency as early as 50 days post-breeding with area under the curve (AUC) values 0.81–0.93^25^.

Pregnancy is a remarkable effectual stage; making an animal acclimatized to certain systemic changes anatomically, physiologically and metabolically to ensure apt fetal development^26^. Precise, timely and uninterrupted delivery of oxygen, nutrients, hormones, and biophysical cues from the mother to fetus is essential for optimum fetal growth^27^. Diligent introspection over the last three decades has vividly revealed that maternal nutrition perturbation (under/over) can affect the fetal growth and development in the intrauterine environment^28^. Further, early embryonic and foetal losses in high-producing bovines has been enumerated to be around 56%. Subsequently, by day 42 of pregnancy, conception and implantation in bovine (cow and buffaloes) is successfully completed and the embryonic mortality falls below 5% afterwards^29^. This clearly urges in-depth introspection into the events of maternal metabolic reprogramming with more emphasis towards the early stage of pregnancy. Because the maternal metabolite trajectory can be valuable for evaluating foetal growth alongside feto-maternal associations, interactions and associated physio-pathological alterations^24,30^. The unearthed knowledge can extend the scope for metabolite maneuvering through precise maternal management to curtail early pregnancy loss. Despite such profound significance, the knowledge database regarding the maternal metabolite dynamicity in the early stage of pregnancy is inadequate in most of the bovine species, including buffaloes. Urine is a preferred biofluid for metabolite analysis as it provides a non-invasive collection method, serves as the repository of diverse metabolites released from the complex feto-maternal metabolic network and has already facilitated biomarker discovery in a similar context^31^. Besides requiring minimal sample preparation, NMR spectroscopy is an ideal modality for metabolite analysis in biological fluids as it extends a perfect blend of ample robustness, considerable sensitivity along with proficient reliability for urinary metabolomics introspection^32^ and demonstrates differential presence of biomolecules in relation to pathophysiological changes^33^. Thus, the current investigation has been carried out to explicate the maternal urinary metabolite dynamicity during early pregnancy in buffaloes as well as to identify the potential early pregnancy diagnostic metabolomics signatures through NMR analysis in a need-based and timely manner. This may satisfy the quest for metabolic biomarkers of early pregnancy diagnosis as well as deciphering the optimum metabolic profile of healthy pregnancy and elucidating key regulated metabolic pathways during early gestation.

## Results and Discussion

### Identification of the differentially expressed urinary metabolites

A total of twenty-four metabolites were identified through ^1^*H* NMR analysis in the urine samples of pregnant and non-inseminated control animals on different days of estrous cycle/pregnancy (Table 1). Among them, twenty metabolites were consistently detected in the urine samples of pregnant as well as control animals on all the relevant experimental days. The four metabolites: 1,6-Anhydro-β-D-Glucose, Fumarate, Maleate and Tyramine that were not consistently detected in the urine samples of pregnant as well as control animals at all the different experimental days were excluded from univariate statistical analysis. However, Tyramine was exclusively detected in the pregnant animals only after 18 days of pregnancy with a consistently up-regulating trend during the subsequent experimental days. Among the twenty consistently detected urinary metabolites, 1-Methylhistidine, 3-Hydroxykynurenine, 3-Indoxysulfate, Anthranilate, Phenylalanine, Tryptophan, Tyrosine and 5-Hydroxytryptophan were depicted statistically significant (*P* < 0.05, FDR 0.05) differentiating pattern in ANOVA analysis at day 10 and day 18 between pregnant and control animals (Table 1). Moreover, within the pregnant group, the concentrations of these eight metabolites viz*.* 1-Methylhistidine, 3-Hydroxykynurenine, 3-Indoxysulfate, Anthranilate, Phenylalanine, Tryptophan, Tyrosine and 5-Hydroxytryptophan at day 10 and day 18 differed significantly (*P* < 0.05) from their concentrations at 0 day with exceptions in case of 3-Hydroxykynurenine at day 10, Tryptophan at day 10 and Phenylalanine at day 18 where the variations were non-significant from their day 0 value in pregnant animals. However, the filtering the criteria of the differentially expressed metabolites for potential early pregnancy biomarker identification were set as minimum twofold metabolite up/down-regulation from day 18 onwards in pregnant samples with respect to metabolite concentration of control animals at all day points except day 0, along with a consistent trend in metabolite up/down-regulation upto 42 days of pregnancy. The day 0 was escaped as it was the day of estrus, showing the typical receptive behavioural pattern of the animals to easily differentiate from pregnancy. Four differentially expressed metabolites were viz. 3-Hydroxykynurenine, Anthranilate, Tyrosine and 5-Hydroxytryptophan satisfied all the aforesaid criteria of the potential early pregnancy detection biomarker and were subjected to Receiver Operating Characteristic (ROC) curve analyses. Among these four metabolites, anthranilate and 5-Hydroxytryptophan matched all the criteria as well as depicted a prominent up-regulating trend as early as day 10 of pregnancy with an over tenfold increase with respect to their respective day 0 levels. Despite being differentially expressed, 1-Methylhistidine, 3-Indoxysulfate, Phenylalanine, and Tryptophan were excluded from ROC curve analysis as they were not consistently up/down-regulated till 42 days of pregnancy.

**Table 1:** Comparative analysis of the urinary metabolites identified and quantified from pregnant and non-pregnant groups of Murrah Buffalo heifers using NMR Spectrometry.

Name of Metabolite	Status	Level in urine (microMol); day post insemination/estrus				
		0	10	18	35	42
1,6-Anhydro-β -D-Glucose	Preg	27.05 ± 5.17	ND	ND	1,741.6 ± 78.72	1,467.05 ± 81.59
	NP	40.93 ± 6.01	95.40 ± 5.22	39.90 ± 5.13	–	–
1-Methylhistidine	Preg	84.58^ax^ ± 0.95	757.68^bx^ ± 46.06	1,070.41^cx^ ± 48.52	248.10^d^ ± 9.68	779.48^b^ ± 121.35
	NP	95.24^ay^ ± 1.79	426.35^by^ ± 20.29	753.28^cy^ ± 32.00	–	–
3-Hydroxykynurenine	Preg	1,023.83^a^ ± 37.60	1,091.81^ax^ ± 93.65	1,481.46^bx^ ± 10.88	1,671.86^c^ ± 24.98	1,938.16^d^ ± 25.70
	NP	1,004.81^a^ ± 24.54	749.83^by^ ± 42.31	451.00^cy^ ± 12.61	–	–
3-Indoxysulfate	Preg	176.78^a^ ± 22.81	1,083.88^bx^ ± 15.65	874.86^cx^ ± 35.94	651.06^d^ ± 102.71	248.36^a^ ± 16.20
	NP	152.93^a^ ± 21.86	347.0^ay^ ± 38.81	591.43^by^ ± 29.84	–	–
Acetate	Preg	190.5 ± 2.40	682.58 ± 71.98	287.01 ± 23.47	509.93 ± 96.67	493.70 ± 80.28
	NP	522.81 ± 358.93	262.63 ± 32.16	162.91 ± 14.50	–	–
Anthranilate	Preg	25.66^a^ ± 0.21	616.3^bx^ ± 18.08	909.86^cx^ ± 17.72	1,336.53^d^ ± 31.19	2,018.70^e^ ± 72.34
	NP	22.22^a^ ± 0.92	315.15^by^ ± 20.06	199.31^by^ ± 10.34	–	–
1,3-Dihydroxyacetone	Preg	61.91^a^ ± 9.40	418.70^b^ ± 62.78	856.38^cx^ ± 77.67	278.63^ab^ ± 38.23	432.91^b^ ± 81.59
	NP	63.85^a^ ± 7.22	617.96^b^ ± 46.08	213.48^ay^ ± 12.10	–	–
Chlorogenate	Preg	210.18^a^ ± 6.14	667.5^a^ ± 117.65	1,034.26^bx^ ± 13.60	190.20^ac^ ± 40.50	1,235.95^b^ ± 267.64
	NP	206.15 ± 10.67	217.73 ± 14.93	334.41^y^ ± 32.59	–	–
EthyleneGlycol	Preg	151.30^a^ ± 14.38	1,179.75^bx^ ± 193.32	516.56^c^ ± 18.03	337.20^ac^ ± 15.56	187.11^a^ ± 27.46
	NP	148.30^a^ ± 11.71	212.56^ay^ ± 14.91	611.06^b^ ± 18.52	–	–
Fumarate	Preg	119.86 ± 11.87	ND	ND	190.20 ± 12.99	80.75 ± 7.43
	NP	95.93 ± 6.33	200.90 ± 11.17	233.75 ± 16.19	–	–
Glycolate	Preg	104.56^a^ ± 5.76	7,203.95^bx^ ± 480.40	1,362.43^c^ ± 118.16	7,512.48^b^ ± 544.73	551.13^ac^ ± 55.43
	NP	90.76 ± 9.65	184.71^y^ ± 24.16	601.05 ± 32.66	–	–
Leucine	Preg	37.55^a^ ± 8.60	163.73^bx^ ± 33.26	93.48^ac^ ± 10.07	167.58^b^ ± 4.71	142.90^cb^ ± 18.00
	NP	30.30 ± 4.76	80.50^y^ ± 9.24	96.25 ± 9.31	–	–
Melatonin	Preg	184.03^ab^ ± 20.10	202.73^ac^ ± 41.69	245.75^acx^ ± 32.39	165.48^ab^ ± 11.61	97.01^ab^ ± 7.06
	NP	164.68 ± 17.78	139.40 ± 12.20	70.48^y^ ± 9.25	–	–
Maleate	Preg	43.98 ± 7.08	ND	ND	671.28 ± 45.64	1,106.28 ± 129.13
	NP	51.60 ± 10.91	226.56 ± 39.20	450.15 ± 36.79	–	–
Phenylalanine	Preg	1,623.46^a^ ± 25.38	2,185.03^bx^ ± 58.18	1,710.83^ax^ ± 17.29	284.18^c^ ± 16.89	195.91^c^ ± 8.91
	NP	1,624.55 ± 15.15	1,567.69^y^ ± 19.37	1,506.54^y^ ± 28.49	–	–
Protocatechuate	Preg	531.38^a^ ± 17.65	438.53^abx^ ± 11.61	415.66^b^ ± 38.96	142.05^c^ ± 11.42	112.30^c^ ± 4.18
	NP	509.90^a^ ± 24.11	662.45^by^ ± 26.32	362.80^c^ ± 29.06	–	–
Quinolinate	Preg	42.62^a^ ± 1.38	413.05^a^ ± 22.98	575.53^a^ ± 50.80	761.96^a^ ± 19.33	1,116.46^b^ ± 32.34
	NP	650.52 ± 1.07	499.55 ± 26.56	550.96 ± 35.25	–	–
Serotonin	Preg	21.09^a^ ± 0.30	250.30^bx^ ± 18.52	432.80^c^ ± 25.19	859.50^d^ ± 37.15	1,045.96^e^ ± 54.59
	NP	18.32^a^ ± 0.21	581.96^by^ ± 25.92	309.80^c^ ± 34.36	–	–
Tryptophan	Preg	290.08^a^ ± 9.68	338.96^abx^ ± 21.11	454.35^bx^ ± 43.67	176.90^a^ ± 21.15	213.41^a^ ± 21.09
	NP	270.25^a^ ± 17.68	640.30^by^ ± 37.88	303.03^ay^ ± 26.32	–	–
Tyrosine	Preg	87.70^a^ ± 10.21	371.18^bx^ ± 25.29	314.73^bx^ ± 7.89	530.58^c^ ± 15.87	816.13^d^ ± 26.26
	NP	91.03^a^ ± 8.06	120.71^aby^ ± 5.73	164.71^by^ ± 15.74	–	–
5-Hydroxytryptophan	Preg	37.06^a^ ± 8.97	796.35^bx^ ± 27.59	931.33^bx^ ± 30.78	1,804.15^c^ ± 92.91	2,174.05^d^ ± 44.60
	NP	42.11^a^ ± 3.97	416.48^by^ ± 19.21	219.95^ay^ ± 19.29	–	–
Histidine	Preg	223.38^a^ ± 21.05	483.98^bx^ ± 41.54	181.93^ac^ ± 18.55	97.16^c^ ± 8.06	53.08^c^ ± 6.87
	NP	205.71^a^ ± 21.71	756.15^by^ ± 39.99	190.05^a^ ± 12.42	–	–
Valerate	Preg	51.26^a^ ± 11.50	607.43^bx^ ± 94.14	453.00^c^ ± 35.48	597.76^bc^ ± 72.34	791.45^b^ ± 20.20
	NP	59.36^a^ ± 4.75	168.21^aby^ ± 20.62	372.70^b^ ± 30.35	–	–
Tyramine	Preg	ND	ND	147.17 ± 16.36	683.33 ± 18.60	714.83 ± 21.05
	NP	ND	ND	ND	–	–

Mean with different superscripts (a, b, c and d) within the row i.e. between different days of pregnancy and (x, y) between the row i.e. between pregnant and non-pregnant on same days for a particular group differ significantly (*p* < 0.05). Preg. (pregnant), NP (non-pregnant) and ND (not detected).

### Multivariate analysis of the urine metabolites

**Figure 1:**
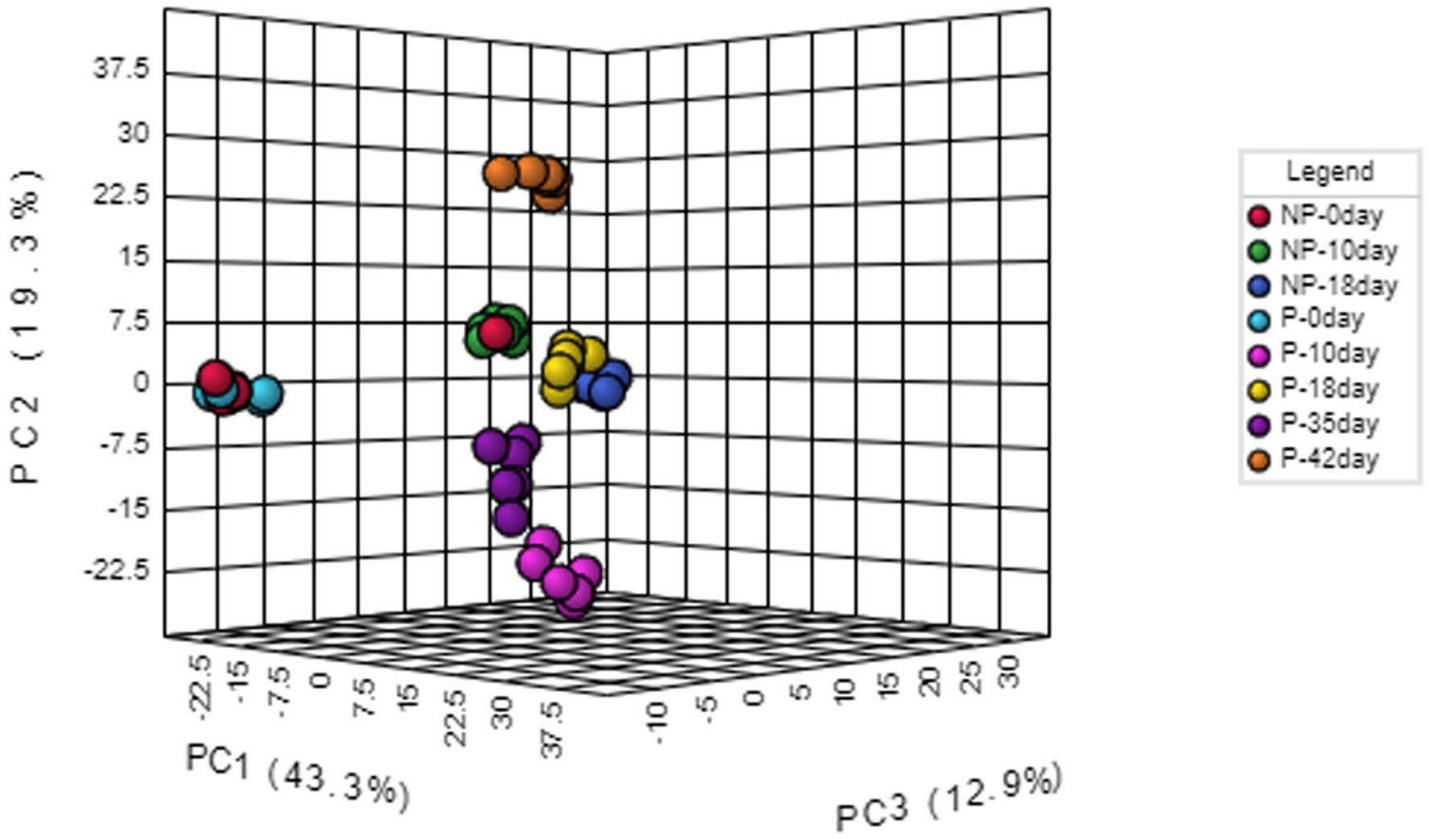
Principal component analysis (PCA) of the five principal components PC1 (43.3%), PC2 (19.3%), PC3 (12.9%), PC4 (9.6%) and PC5 (5.8%) revealing the overall metabolic differences between the pregnant and non-pregnant samples at different time-points (day 0, day 10, day 18, day 35 and day 42).

**Figure 2:**
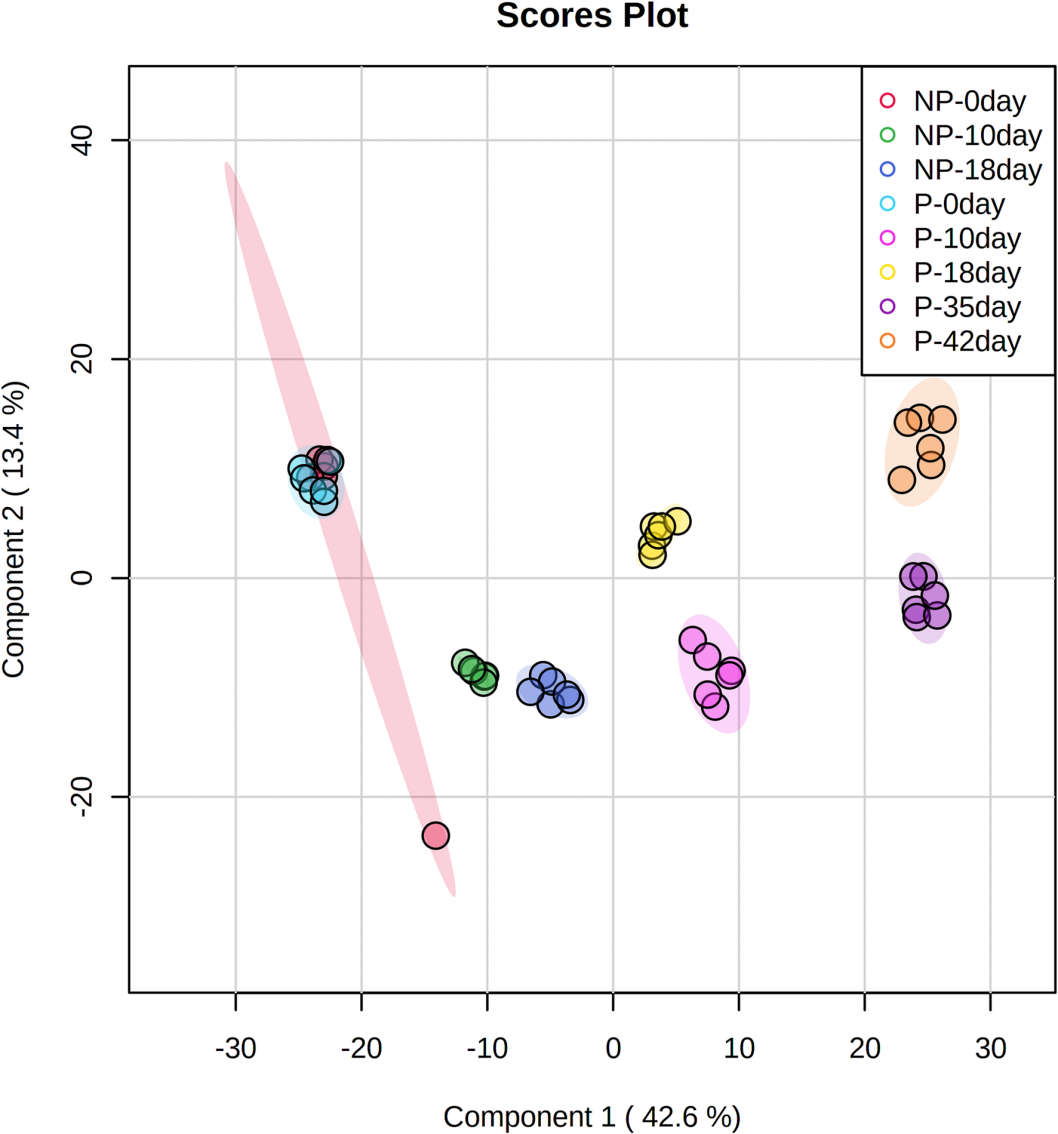
The 2D score plot of PLS-DA analysis incorporating Component 1 (42.6%) and Component 2 (13.4%) depicting prominent separate clusters of inter-group as well as inter-day variations except at day 0.

**Figure 3:**
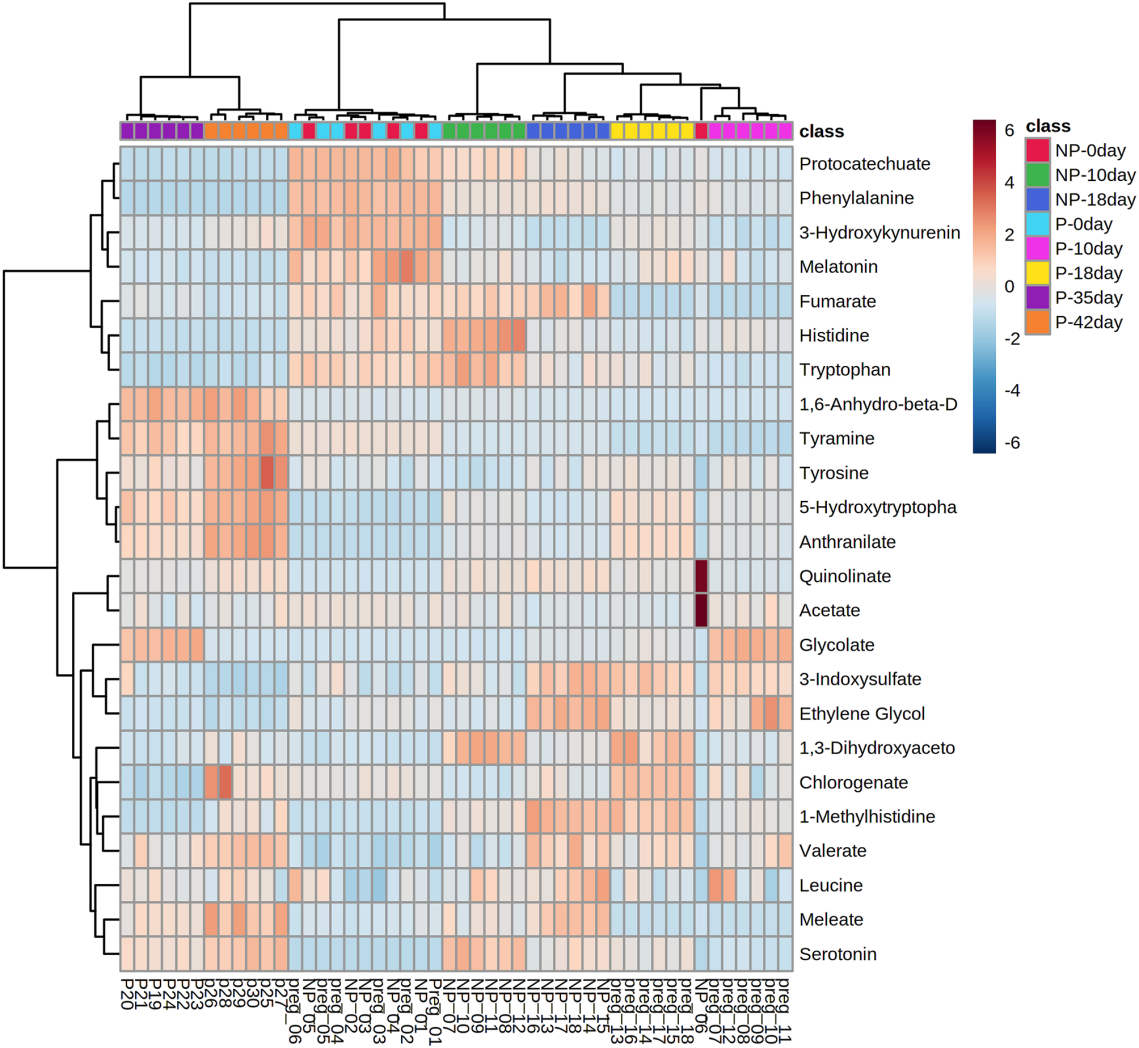
Hierarchical clustering of the differentially expressed metabolites depicted in the heat map. Only metabolites from the day 0 samples of pregnant (P) and non-pregnant (NP) group mingled with each other while the metabolites of the other group-specific samples at different days orient them in different distant clads in the dendrogram.

**Figure 4:**
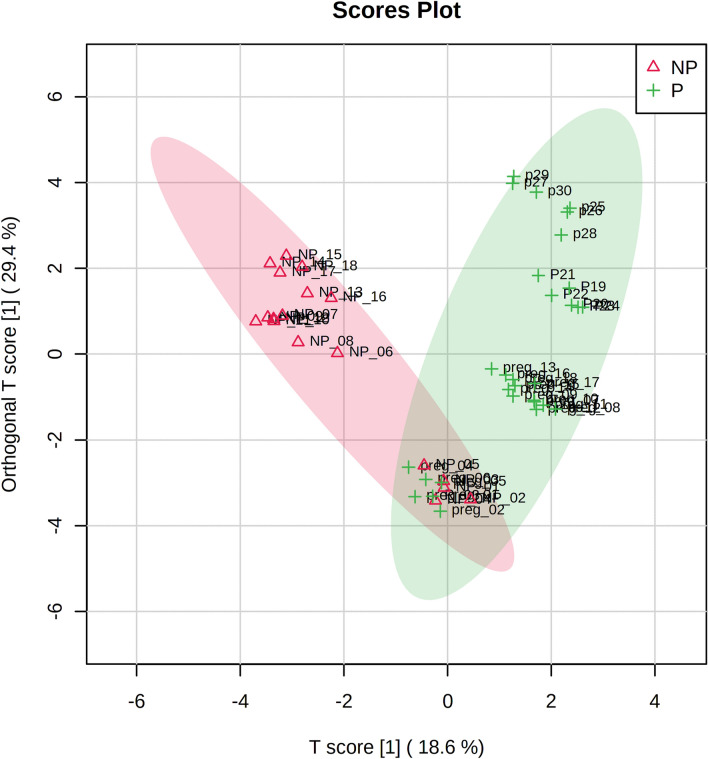
Score plot of OPLS-DA analysis depicting prominent variations in metabolite profile between the pregnant (P) and non-pregnant (NP) samples; the superimposed area of both the ellipse indicated the overlapped metabolite profile of the two groups at the day 0 time-point.

**Figure 5:**
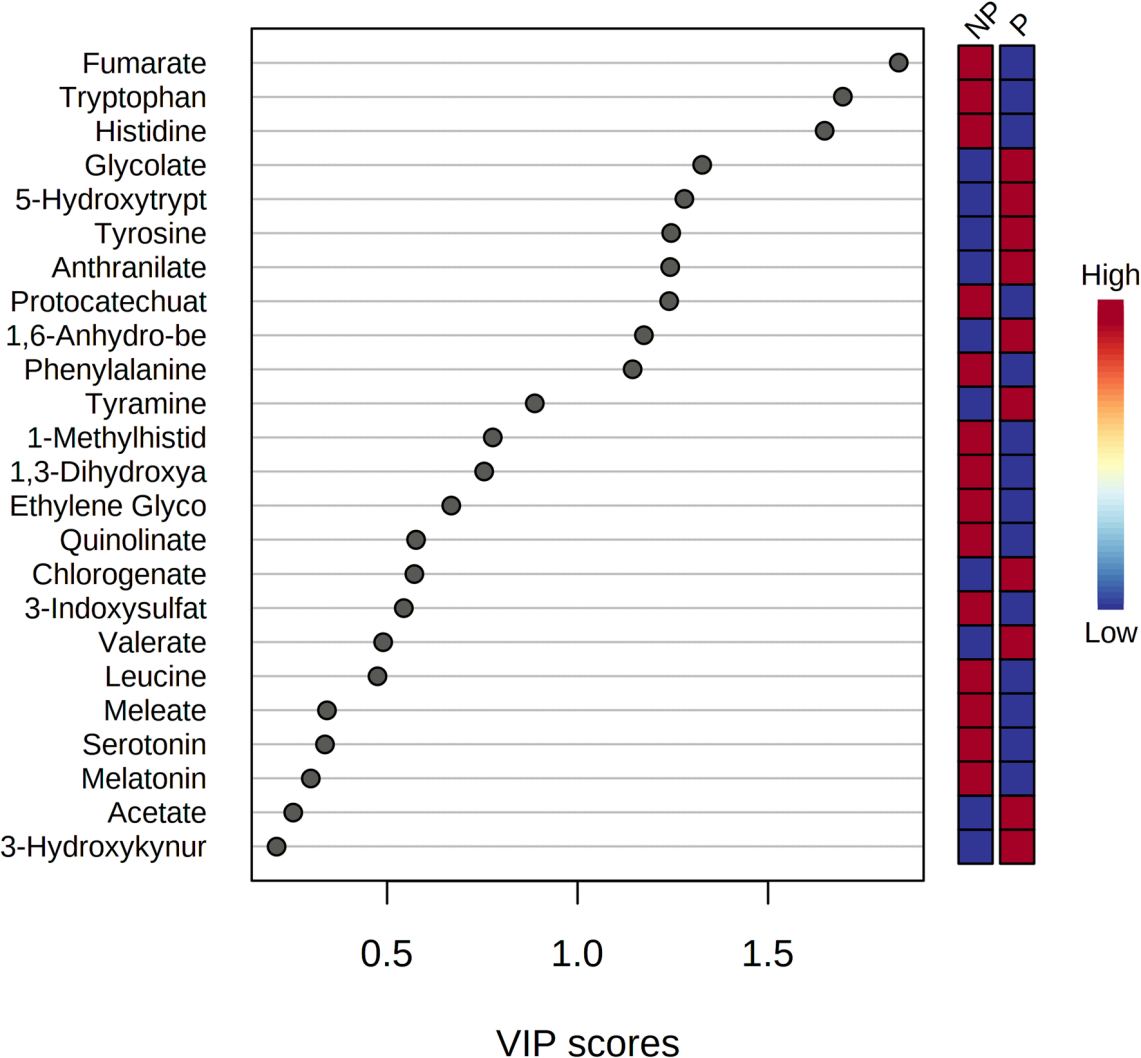
Variable Importance in Projection (VIP) score of the metabolites through OPLS-DA analysis; as potential early pregnancy detection biomarkers 5-hydroxytryptophan, Tyrosine and Anthranilate showed VIP score above 1 and depicted high abundance ratio in pregnant samples (corresponding heat-map).

Principal component analysis (PCA) employed five principal components, PC1 (43.3%), PC2 (19.3%), PC3 (12.9%), PC4 (9.6%) and PC5 (5.8%) to elucidate the overall metabolic differences between the pregnant and non-pregnant samples at different time-points (day 0, day 10, day 18, day 35 and day 42). In PC1 vs PC2 vs PC3 analysis, samples from the pregnant and non-pregnant animals overlap only at day 0 (denoted as P-0 day and NP-0 day, respectively in Fig. 1); while the pregnant animal samples at day 10, day 18, day 35 and day 42 (denoted as P-10 day, P-18 day, P-35 day, P-42 day, respectively in Fig. 1) as well as non-pregnant animal samples at day 10 and day 18 (denoted as NP-10 day and NP-18 day, respectively in Fig. 1) segregated as different clusters that clearly depicted inter-group as well as inter-day variations in metabolite profile in PCA synchronized 3D plot. One of the non-pregnant females at day 0 clustered with all the samples from non-pregnant females on day 10 as evidenced from Fig. 1 may be due to the individual variations appearing from the inclusion of buffaloes having estrous cycles with 1, 2 or 3 follicular waves in the same group. Although 2-wave cycles are the most usual, but animals with a 1-wave or 3-wave follicular growth pattern also exists and influences the length of the luteal phase as well as the estrous cycle^34^. Further, variability in follicular phase is also common to buffaloes, like shy breeders might underline such individual variation^35^. The 2D score plot of PLS-DA analysis incorporating Component 1 (42.6%), Component 2 (13.4%) also presented a similar pattern as in PCA analysis, depicting prominent separate clusters of inter-groups as well as inter-day variations except on day 0 (Fig. 2). The hierarchical clustering of the differentially expressed metabolites depicted in the heat map also represented that metabolites only in the day 0 samples of pregnant and non-pregnant animals mingled with each other, while the metabolites in the other group as well as the day-specific samples orient them in different distant clads in the dendrogram (Fig. 3). Further, the score plot of the OPLS-DA analysis represented more prominent variation in the metabolite profile between pregnant and the non-pregnant samples, while the superimposed area of both the ellipses indicated the overlapped metabolite profile of the pregnant and the non-pregnant samples at the day 0 time-point (Fig. 4). Permutation validation suggested no over fitting of the OPLS-DA model with Q2 of 0.606 and R2Y of 0.691, *p* < 0.01. Variable Importance in Projection (VIP) score of the metabolites through OPLS-DA analysis elucidated that 5-hydroxytryptophan (1.28094), Tyrosine (1.2463) and Anthranilate (1.24374) were having VIP score above 1 and depicted high abundance ratio in pregnant samples (corresponding heat-map) among the four differentially expressed metabolites identified as the potential early pregnancy detection biomarker through ANOVA (Fig. 5).

**Figure 6:**
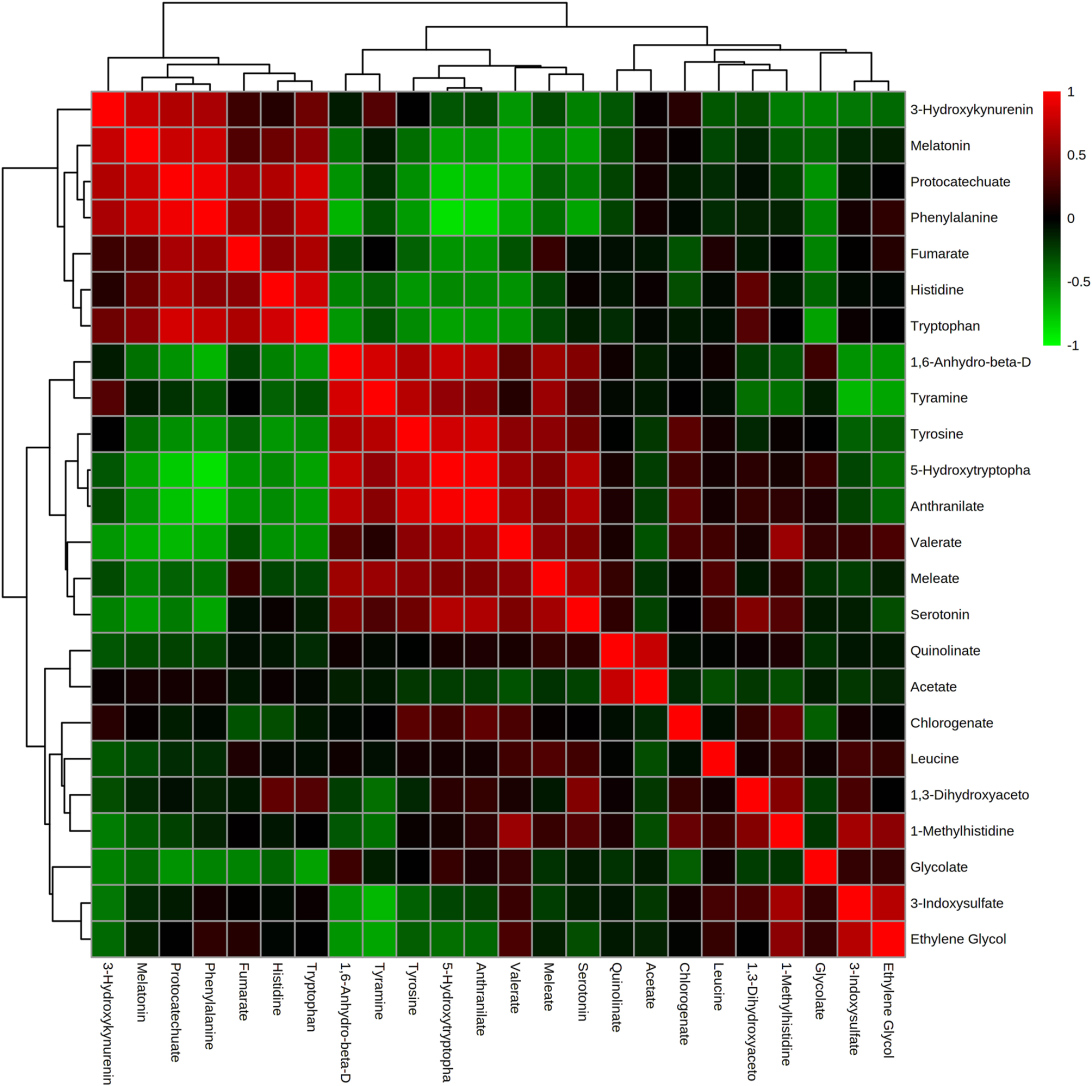
Correlation analyses of the differentially expressed metabolites depicting different degree of association among them (denoted with the color ladder at top-right).

Further, correlation analyses among the four differentially expressed potential metabolite biomarkers depicted that 5-Hydroxytryptophan was positively correlated with Tyrosine (*r* = 0.80956; *P* < 0.001) and Anthranilate (*r* = 0.97457; *P* < 0.001) while inversely correlated with 3-Hydroxykynurenine (*r* = − 0.33014; *P* = 0.021927). Evidently, 3-Hydroxykynurenine depicted a non-significant correlation with Tyrosine (*r* = 0.00041668; *P* = 0.99776) and inverse correlation with Anthranilate (*r* = − 0.29251; *P* = 0.043641) (Fig. 6). Serotonin is a key metabolite of tryptophan metabolism, was found to be positively correlated with Anthranilate (*r* = 0.67772; *P* < 0.001) and 5-Hydroxytryptophan (*r* = 0.70339; *P* < 0.001) while inversely correlated with 3-Hydroxykynurenine (*r* = − 0.49279; *P* < 0.001). Quinolinate, another product of tryptophan metabolism, depicted an inverse correlation with 3-Hydroxykynurenine (*r* = − 0.32987; *P* = 0.022042) and non-significant correlation with Anthranilate (*r* = 0.1152; *P* = 0.43558) and 5-Hydroxytryptophan (*r* = 0.095816; *P* = 0.5171). Whereas Tryptophan was depicted with a weak positive correlation with 3-Hydroxykynurenine (*r* = 0.42364; *P* = 0.0026965) and a strong inverse correlation with Anthranilate (*r* = − 0.60769; *P* < 0.001) and 5-Hydroxytryptophan (*r* = − 0.62684; *P* < 0.001) (Fig. 6).

### Analysis of predictive ability of the potential biomarkers

**Figure 7:**
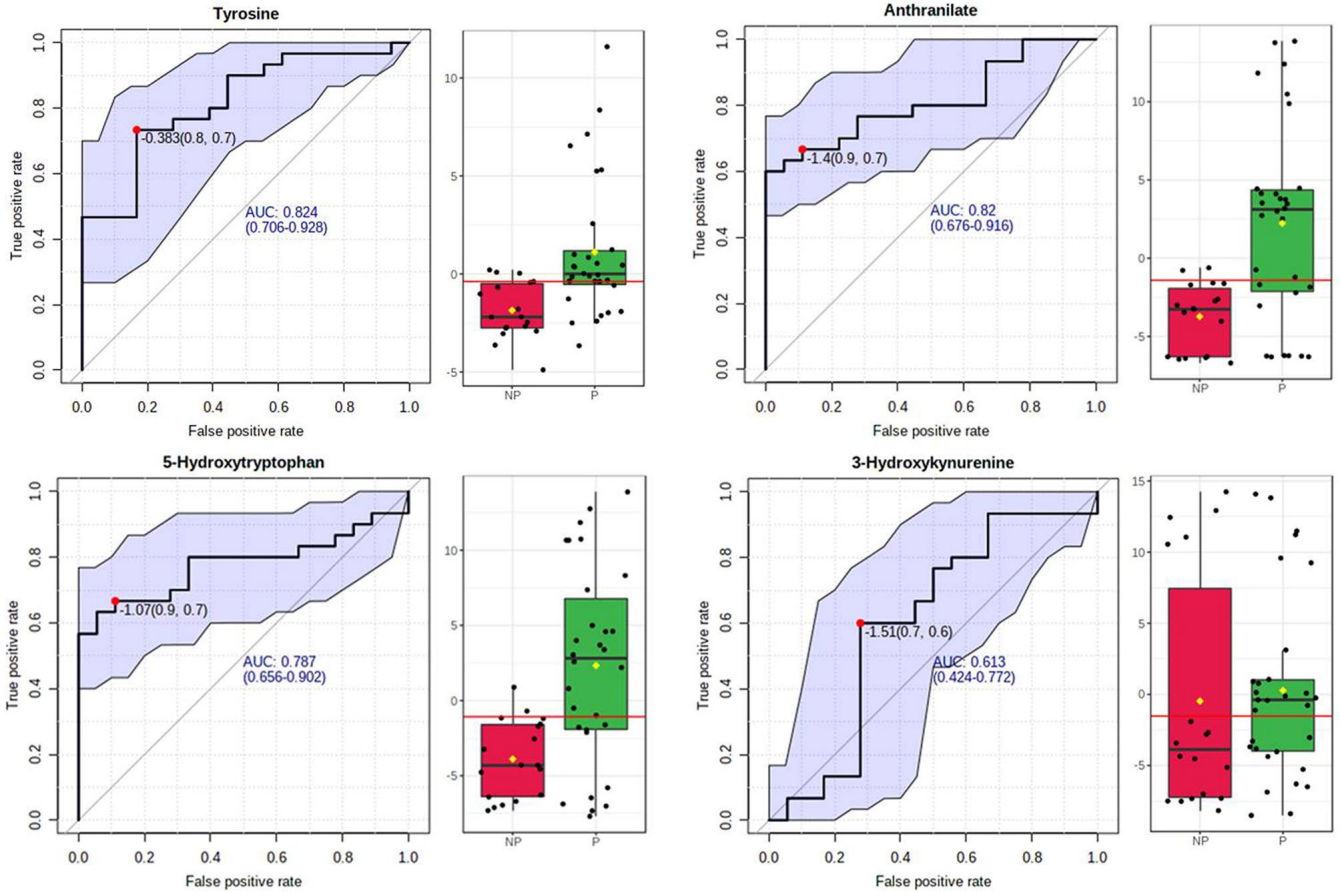
The predictive ability of the individual potential biomarkers based on ROC AUC values along with with Sensitivity and Specificity at 95% CI. The black dots in the boxplot of the selected metabolite (at right side) represented its’ concentrations in all samples. The notches indicated difference in the selected metabolite concentration between the groups; if the notches did not overlap, the medians were likely different. The mean concentration of each group was denoted with a yellow diamond over lying on the respective notch. The optimal cutoff was indicated with a horizontal red line on the boxplot.

**Figure 8:**
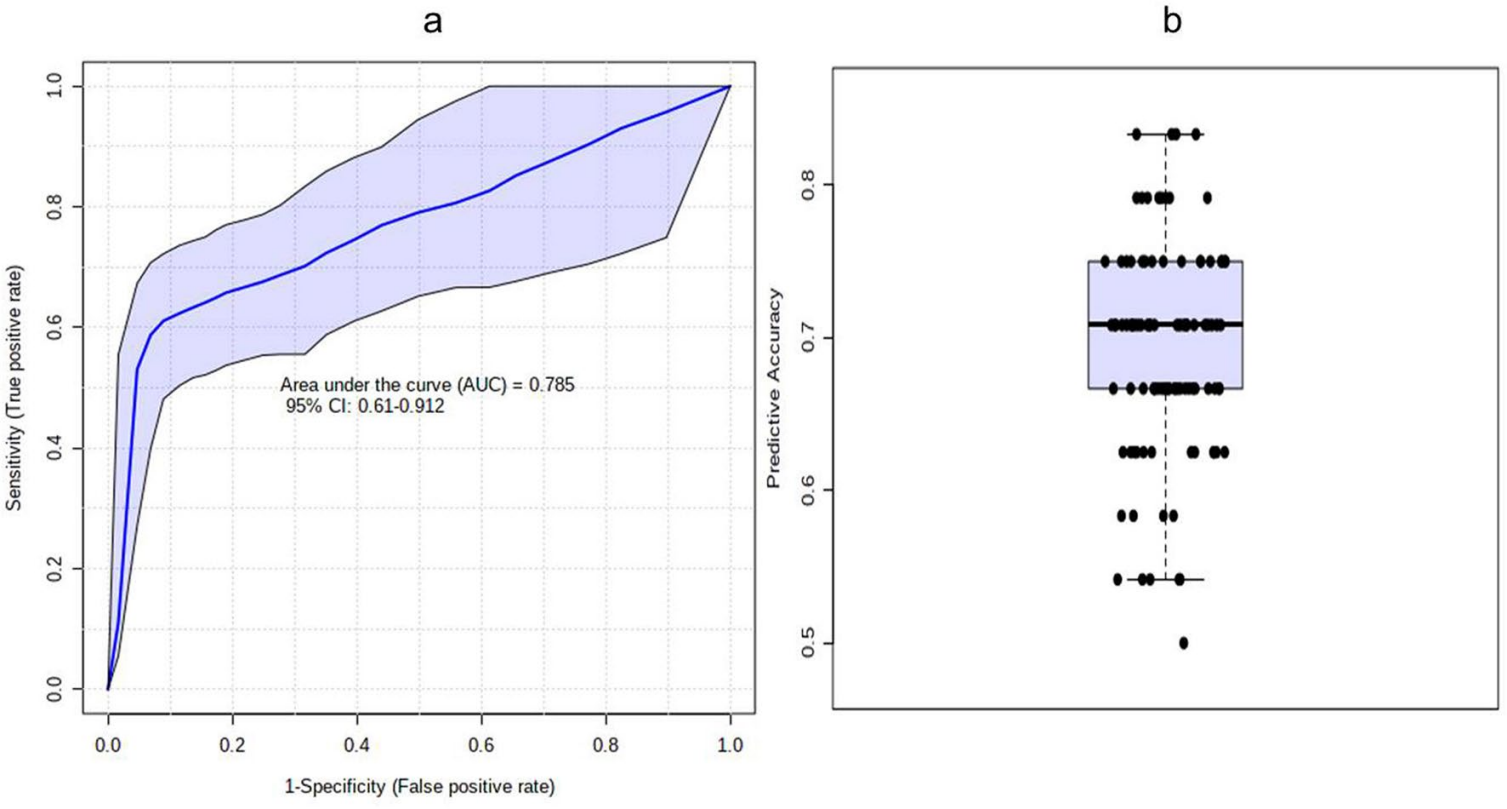
(**a**) The predictive ability of the combination of Anthranilate, 3-Hydroxykynurenine, Tyrosine, and 5-Hydroxytryptophan based on ROC AUC values along with with Sensitivity and Specificity at 95% CI. (**b**) The average predictive accuracy of the metabolite combination based on 100 cross validations.

**Figure 9:**
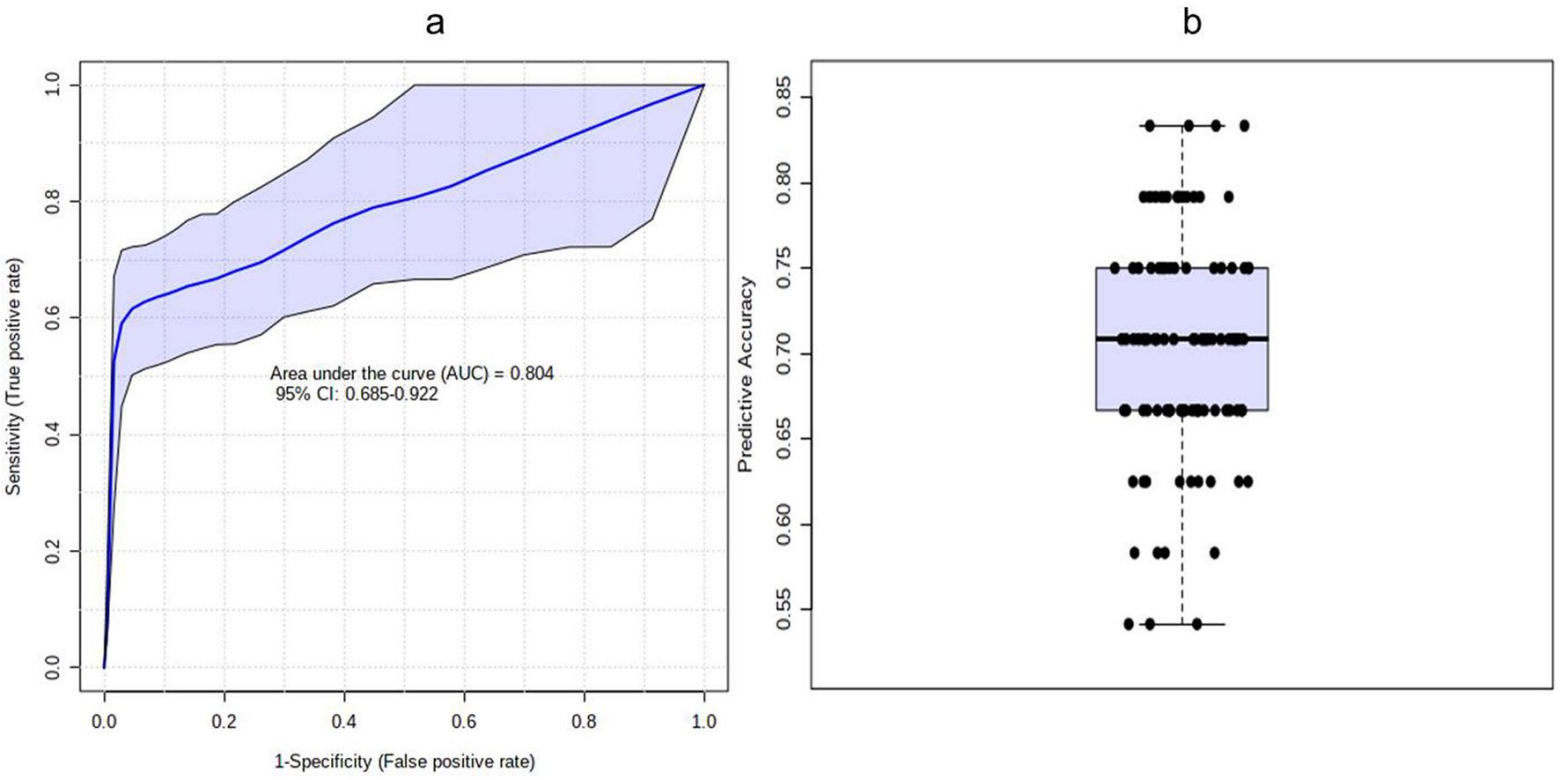
(**a**) The predictive ability of the combination of Anthranilate, 3-Hydroxykynurenine, and Tyrosine based on ROC AUC values along with with Sensitivity and Specificity at 95% CI. (**b**) The average predictive accuracy of the metabolite combination based on 100 cross validations.

**Figure 10:**
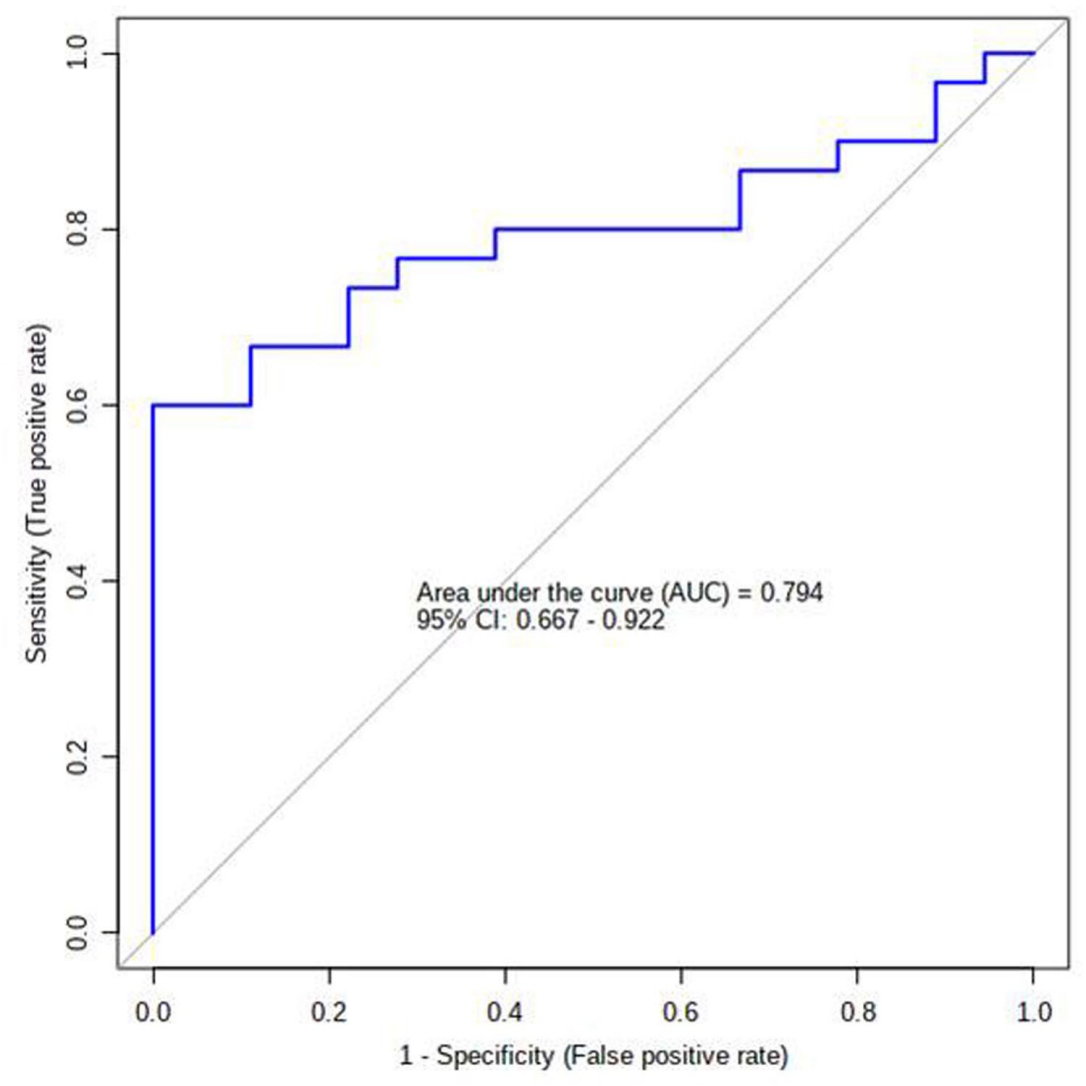
The performance of the logistic regression model constructed by using the combination of Anthranilate, 3-Hydroxykynurenine, and Tyrosine based on ROC AUC values along with with Sensitivity and Specificity at 95% CI in tenfold Cross Validation.

The predictive ability of the individual potential biomarkers as identified through ANOVA was analysed by calculating their Receiver Operating Characteristic (ROC) area under the curve (AUC) value through classical univariate ROC curve analysis. The ROC AUC values were 0.824 for Tyrosine with Sensitivity: 0.733 (0.583–0.867) and Specificity: 0.833 (0.638–1) at 95% confidence interval (CI); 0.82 for Anthranilate with Sensitivity: 0.667 (0.5–0.8) and Specificity: 0.889 (0.722–1) at 95% CI; 0.787 for 5-Hydroxytryptophan with Sensitivity: 0.667 (0.432–0.867) and Specificity: 0.889 (0.722–1) at 95% CI and 0.613 for 3-Hydroxykynurenine with Sensitivity: 0.6 (0.449–0.767) and Specificity: 0.722 (0.556–0.889) at 95% CI (Fig. 7 and Table 2). The possibility of improvement in the prediction efficiency was also verified by employing a combination of more than one manually selected discriminatory metabolite via logistic regression analysis. However, a combination of the four features, viz. Anthranilate, 3-Hydroxykynurenine, Tyrosine, and 5-Hydroxytryptophan (the frequency % in LASSO modeling were 100, 80, 40, and 20 respectively) did not improve the predictive ability yielding ROC-AUC value of 0.785, 95% CI: 0.61–0.912 (Fig. 8a). The average predictive accuracy of the metabolite combination was found to be 0.691 based on 100 cross validations (Fig. 8b). The best ROC-AUC value of 0.804, 95% CI: 0.685–0.922 was achieved by combining Anthranilate, 3-Hydroxykynurenine, and Tyrosine with average predictive accuracy of 0.703 based on 100 cross validations (Fig. 9a and b). A logistic regression (LR) model was derived with the three selected compounds using the tenfold Coss Validation. The equation of the LR model: logit(P) = log(P/(1 − P)) = 2.103 + 0.146 3-Hydroxykynurenine + 0.402 Tyrosine + 0.517 Anthranilate, where the numeric value of each named metabolite in the equation is the concentration after log transformation and auto-scaling. The performance of the LR Model in tenfold Cross Validation yielded an AUC value of 0.794, 95% CI: 0.667–0.922, Sensitivity: 0.733 (0.733 ~ 0.892) and Specificity: 0.778 (0.586 ~ 0.970) (Fig. 10).

**Table 2:** Predictive ability of the individual potential biomarkers based on ROC curve analysis.

Metabolite	P-value	Fold Change	log2 (FC)	ROC-AUC value
Tyrosine	0.0015266	0.2959	− 1.7567	0.824
Anthranilate	0.000348184	0.1823	− 2.4557	0.82
5-Hydroxytryptophan	0.00035073	0.1969	− 2.3443	0.787
3-Hydroxykynurenine	0.73042	0.5101	− 0.97126	0.613

### Metabolic pathway impact and functional analysis

**Figure 11:**
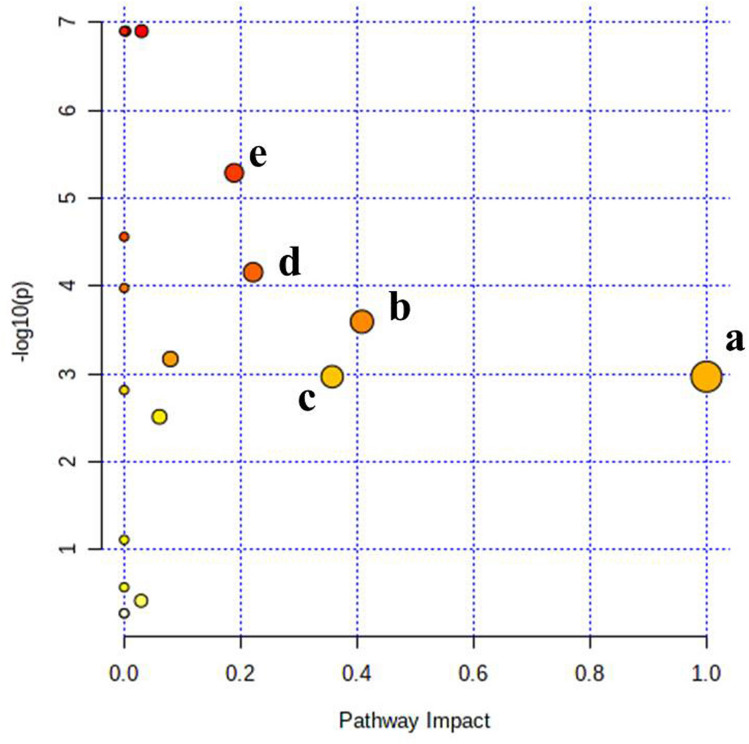
Summary of the metabolic pathway analysis and their impact on pregnancy with MetaboAnalyst 5.0. (**a**) Phenylalanine, tyrosine and tryptophan biosynthesis, (**b**) Tryptophan metabolism, (**c**) Phenylalanine metabolism, (**d**) Histidine metabolism, (**e**) Tyrosine metabolism.

**Figure 12:**
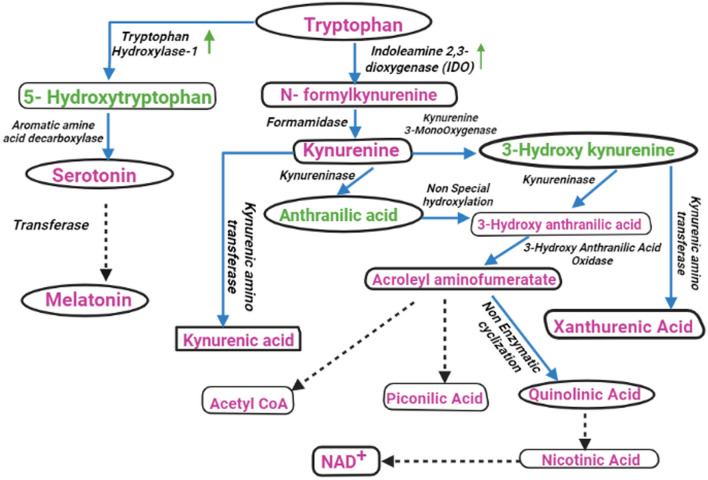
Potential pathway modulations of tryptophan metabolism and the derived metabolites during early gestation in Murrah buffaloes. The figure is created in BioRender.com.

Based on the urinary metabolite profile of pregnant and non-pregnant samples, metabolic pathway analysis was performed using MetaboAnalyst 5.0 to elucidate the most relevant pathways modulated in response to pregnancy. The impact value of those pathways above 0.1 derived from pathway topology analysis was identified as the most potent pregnancy-associated pathway modulations. According to the impact values, five metabolic pathways, *vizly* Phenylalanine, tyrosine and tryptophan biosynthesis, Tryptophan metabolism, Phenylalanine metabolism, Histidine metabolism and Tyrosine metabolism were identified as the most relevant pathways to be regulated in the early stage of pregnancy (Fig. 11). Further, the pathway analysis also depicted these five pathways encompassing several key metabolites that were identified in the current study and also held different levels of significance in pathway modulation, such as Phenylalanine, Tyrosine, Tryptophan, Anthranilate, 5-Hydroxytryptophan, Serotonin, Melatonin, Histidine, 1-Methylhistidine, Tyramine, Fumarate, etc. and significantly, the potential biomarkers predicted in the current study were also in accordance with the observation (Table 3) (Figure S1–S5). In coherence with our finding, pregnancy-associated plasma metabolite alteration due to modulation in phenylalanine, tyrosine and tryptophan biosynthesis pathways was also reported in pregnant multiparous holestein cows during early gestation^36^. This is also very well-correlated with the observations in correlation analysis of these potential metabolite biomarkers. Anthranilate, 3-Hydroxykynurenine, and 5-Hydroxytryptophan are the products of tryptophan metabolism which is keenly associated with successful completion of mammalian pregnancy because of (i) increased maternal demand, (ii) fetal growth and development, (iii) involvement in serotonin for signaling pathways, (iv) kynurenic acid (KA) for neuronal protection, (v) quinolinic acid for NAD^+^ synthesis, (vi) other kynurenines (Ks) for suppressing fetal rejection^37–39^ (Fig. 12). The metabolic pathway modulations as well as urinary detection of phenylalanine, tyrosine and the products of the tryptophan metabolism such as 3-hydroxykynurenine, 5-hydroxytryptophan, anthranilate, quninolate, serotonin and melatonin depicted in the current study was also in consonance with the findings of metabolomics introspection in holestein cows during early gestation where tyrosine metabolism was reported to be modulated on day 17 and day 45 of pregnancy while phenylalanine, tyrosine and tryptophan biosynthesis was reported to be altered on day 45 of pregnancy^36^. Probably, elevation in tryptophan utilization takes place during pregnancy yielding several derivatives as well as certain organic acids through the serotonin pathway and the kynurenine pathway. Concentration of 5-hydroxytryptophan, a serotonin pathway intermediate, was found to be increased in fetal cotyledons of buffaloes with advancement of pregnancy^40^. Further, tryptophan hydroxylase-1 expression was found to be induced by pregnancy through lactogenic signaling resulting in elevated synthesis of 5-hydroxytryptophan in pancreatic islets that promoted insulin producing beta cell proliferation (Fig. 12). Thus, elevated production of 5-hydroxytryptophan and subsequent serotonin synthesis prevent maternal hyperglycemia and modulate energy metabolism to accommodate the foetal burden^41^. Melatonin, the downstream product of the serotonin pathway, was also observed to increase the expression of antioxidant enzymes in placenta^42^, improves placental efficiency, birth weight of the foetus and reduces oxidative and hypoxic stress^43^. So, enhanced tryptophan utilization through the serotonin pathway possibly exerted a positive effect on pregnancy establishment and progression in buffaloes. Kynurenine pathway is another principal route of tryptophan metabolism which is associated with immune regulation and providing a tolerogenic environment in the placenta, inducing vasodilation, neovascularization at the feto-maternal surface and regulating oxygen homeostasis^44^. Therefore, the kynurenine pathway holds paramount importance to facilitate establishment and progression of healthy pregnancy, particularly during early gestation by preventing fetal rejection as well as facilitating nutrient supply to the fetus and providing anti-oxidant response by the pathway enzymes and metabolites (e.g. 3-hydroxykynurenine, xanthurenic acid, 3-hydroxyanthranilic acid, and kynurenic acid) in the placental micro-environment^44^. The degree of relevance of the pathway in pregnancy establishment and progression can be justified by the instance that blocking the first and rate-limiting enzyme of the pathway indoleamine 2,3-dioxygenase (IDO) by an IDO-inhibitor 1-methyltryptophan at the onset of pregnancy led to fetal loss in mice while the treatment after pregnancy establishment resulted various pregnancy complications^45–48^. Anthranilate and 3-Hydroxykynurenine, the two potential pregnancy diagnostic biomarkers depicted in the current study, are kynurenine pathway metabolites; evidently, elevated urinary concentration of these metabolites in pregnant samples might be obvious due to induction of kynurenine pathway influenced by strong placental IDO expression during pregnancy (Fig. 12)^47,49,50^. An elevation in urinary concentration of tyrosine in pregnant samples that was depicted as another potential pregnancy diagnostic biomarker in the current study might be due to reduction in tyrosine metabolism and pregnancy associated selective aminoaciduria as reported during early gestation^26,51^. Decreased urinary tyrosine output was also reported to be associated with fetal growth restriction^22^. Further, reduced tyrosine level in maternal circulation was reported to be beneficial for pregnancy as high doses of tyrosine can lower serum progesterone level, resulting in fetal loss in mice^52^. Reduction in circulatory tyrosine also prevents downstream catecholamine production and negates the probability of uterine contraction associated with pregnancy loss^36^. Tyrosine can either be converted to L-DOPA by tyrosine hydroxylase and subsequently to catecholamines or may produce tyramine by tyrosine decarboxylase. As catecholamine over-production is derogatory to sustain the pregnancy, hence, there is a maximum probability of elevation in tyramine production and its urinary output. The exclusive detection of tyramine in the pregnant animals only after 18 days with a persistent upward trend during the subsequent period in the current study also indicated the same.

**Table 3:** Results of pathway analysis with MetaboAnalyst 5.0 indicating potential metabolic pathways to be regulated in the early stage of pregnancy.

Pathway Name	*P* value	Impact	FDR	Relevant metabolites
Phenylalanine, tyrosine and tryptophan biosynthesis	0.0010745	1.0	0.0017583	Phenylalanine, tyrosine, tryptophan,
Tryptophan metabolism	2.5426E-4	0.408	5.7207E-4	Melatonin, Serotonin, Quinolinate, Anthranilate, 3-hydroxykynurenine, 5-Hydroxytryptophan
Phenylalanine metabolism	0.0010745	0.357	0.0017583	Phenylalanine, tyrosine
Histidine metabolism	6.9527E-5	0.221	2.0858E-4	1-methylhistidine, histidine
Tyrosine metabolism	5.1469E-6	0.189	2.3161E-5	Phenylalanine, Tyrosine, Tyramine

So, the outcomes of the present investigation provide the foundation for a novel and accurate urinary metabolite-based early pregnancy diagnosis paradigm in buffaloes to improve the reproductive and productive performance of these animals. Since the metabolomics marker candidates for pregnancy from buffalo urine have been elucidated in the current study, it paves the way to develop specific colorimetric or sensor-based assay for pen-side diagnosis of pregnancy in these animals. The advantage of such diagnostics may be perceived in terms of non-invasive detection, being cost-effective and be performed by farmers without requiring special technical expertise. The current ^*1*^*H* NMR-based biomarker discovery was carried out employing six buffaloes in each group (pregnant and non-pregnant) at different time points that satisfied the criteria of univariate and multivariate statistical analyses in MetaboAnalyst 5.0 program. Similarly, ^*1*^*H* NMR-based metabolomics approach was undertaken to effectively diagnose subclinical ketosis in Holstein cows using six animals each in diseased and control groups^53^. Association between the udder health and milk metabolite profile was also elucidated using NMR spectroscopy employing ten animals divided into two groups based on somatic cell count, each group consisted of five animals^54^. Despite the encouraging results of the initial study, validation of the results in a larger population is advocated for the development of metabolite-based pen-side early pregnancy diagnostics in buffaloes.

## Material and Methods

### Ethical approval

The experiments conducted for the present study were approved by the Institute Animal Ethics Committee (IAEC), Registration no. 406/GO/RBi/L/01/CPCSEA of the Central Institute for Research on Buffaloes, Hisar vide letter IAEC-CIRB/19–20/A/007. The committee works under the supervision of the Committee for the Purpose of Control and Supervision of Experiments on Animals (CPCSEA), which is a statutory Committee of the Department of Animal Husbandry and Dairying, Ministry of Fisheries, Animal Husbandry and Dairying, Government of India constituted under the Prevention of Cruelty to Animals (PCA) Act, 1960. All animals used in the experiment were maintained on scientific lines at the animal farm and were not subjected to any extra discomfort during sample collection for the purpose of this study.

### Animal selection

Apparently, healthy normal cyclic Murrah Buffalo heifers (*n* = 26), that were loose housed and maintained under uniform management conditions at the organised farm of the Central Institute for Research on Buffaloes (CIRB), Hisar, India were employed for the present study. We declare that all methods performed in the current study were in accordance with the ARRIVE guidelines (https://arriveguidelines.org). The heifers were confirmed to be in estrus through visual observations and aided by a teaser bull, were subsequently scanned with ‘B mode’ ultrasound scanner equipped with an intra-operative 7.0 MHz micro-convex multi-frequency transducer for the presence of large dominant ovarian follicle (> 12 mm size). The animals also exhibited good uterine tone and cervical discharge to be selected for the experiment. Twenty (*n* = 20) heifers were inseminated artificially (refereed as AI) using frozen-thawed semen and the remaining six (*n* = 6) animals were maintained as control. The day of estrus/AI was designated as day 0. After insemination, all the animals were monitored for returning to estrus as well as scanned ultrasonographically on days 35 and 42 for ascertaining pregnancy status. From the inseminated animals, eight animals became pregnant, from which six pregnant heifers (n = 6) which sustained healthy pregnancy across day 42 were randomly selected and were designated as ‘pregnant’ group while six non-inseminated normal cyclic animals (*n* = 6) which repeated their estrus cyclicity after 21 days (day 0 of the next cycle) of the last estrus were selected as the ‘non-pregnant’ group. All the methods were performed in accordance with the relevant guidelines and regulations.

### Sample collection and processing

Approximately 250 ml naturally micturated urine samples were collected from all the inseminated animals on days 0, 10, 18, 35 and 42. The animals were retrospectively diagnosed as pregnant through rectal ultrasonography carried out from day 35 onwards. Similarly, the urine samples were collected from the non-inseminated animals only on days 0, 10 and 18 as they returned to estrus after 21 days. The reason behind considering day 0 was to check the status when all animals were non-pregnant while day 10 was chosen in order to get mid diestrus in non-pregnant animals and day 10–18 is the period of conceptus attachment. Further day 18 was taken as sometimes some buffaloes may come in estrous during the period as the cycle mostly varies in the range of 18–21 days. Day 35 was the time point to vividly elucidate the pregnancy status through ultrasonography and day 42 is the stage for completion of implantation and thus substantial reduction in embryonic mortality.

The urine samples were centrifuged and cleaned through 0.45 µM syringe filters (Sigma-Aldrich, USA). These samples were either processed afresh and/or stored at − 80 °C. As pregnancy could only be retrospectively diagnosed by ultrasonography at day 35 level, so, all the samples were stored at − 80 °C only except the day 42 samples that were processed afresh. So, all the samples of other day stage of both the pregnant and non-pregnant group went through similar freezing and processing method. Each urine sample (400 μl) was mixed with a buffer solution (230 μl) containing 0.2% sodium azide (NaN_3_), 0.2 M disodium hydrogen phosphate (Na_2_HPO_4)_, 0.2 M sodium dihydrogen phosphate (NaH_2_PO_4_) and 70 μl of 1 mg/ml sodium 3-trimethylsilyl-(2, 2, 3, 3-D_4_) propionate (TSP) in heavy water (D_2_O) and vortexed for 30 s. The mixture was allowed to stand for 5 min followed by centrifugation at 12,000 × g for 5 min at 4 °C to remove any precipitate. Aliquots of the supernatant (600 μl) were transferred into 5 mm NMR tubes.

### Acquisition of ^1^***H*** NMR spectra

Briefly, ^*1*^*H* NMR spectra of the urine samples were acquired using a Bruker Avance 400 spectrometer (Bruker Biospin, Rheinstetten, Germany) operating at 400.11 MHz and 298 K. The acquisition of ^*1*^*H* NMR spectra of the urine samples were performed during both the recycle delay (1 s) and mixing time (tm, 100 ms) using a standard 1D pulse sequence with water pre-saturation (recycle delay-90°-t1-90°-tm-90°-acquisition; XWIN–NMR 3.5). For each sample, 65,536 Free induction decays (FIDs) were collected into 32 K data points using a 90° pulse length of 13.46 μs with an acquisition time of 4.08 s to obtain 16 scans with a spectral width of 8012.82 Hz. FIDs were zero-filled to twice the size and exponentially multiplied with a line broadening factor of 0.3 Hz before fourier transform. All the ^1^*H* pulse frequency spectra were automatically phase and baseline corrected and calibrated to the peak of TSP (δ 0.00) using TopSpin software version 3.2 (Bruker Biospin, Germany).

### Processing of ^1^***H*** NMR data

All the NMR spectra (spectral region δ 10 to 0.5) were imported into MestReNova software 6.0.2–5475; referenced and corrected for phase and baseline distortion using Mestrelab Research: Analytical Chemistry Software Solutions (Santiago de Compostela, Spain). The spectral regions δ 4.0 to 5.4 were removed prior to the median fold change normalisation as it indicated residual water and urea resonances. Spectral assignments and metabolite quantification were performed using the ‘Profiler and Library Manager’ modules in Chenomx NMR Suite 8.40 (Chenomx Inc, Edmonton, Canada)^55^. Spectral signal from a known concentration of TSP was considered as the reference for metabolite quantification. Additionally, spiking with amino acids in some samples was also performed for further confirmation. Data normalization by sum and Pareto data scaling (mean-centering and division by the square root of standard deviation of each variable) was carried out to minimize the bias arising from samples of different days and replicate variability.

### Univariate statistical analysis

Among the twenty four identified metabolites, twenty metabolites (*n* = 20) which were consistently detected in the urine samples of 6 pregnant as well as 6 non-inseminated control animals at all the relevant experimental days were subjected to univariate analysis by two-way ANOVA using the SPSS software (version 20.0; SPSS, Inc., Chicago, IL, USA)^56^. Data were represented as metabolite concentration ± standard error (SE) and the level of significant differences in metabolite concentrations were considered at *P* < 0.05. The normalized data by sum and Pareto data scaling were used for the statistical analysis to extend equal weightage to all the variables irrespective of their absolute value.

### Multivariate statistical analysis

The metabolite data normalized by sum and Pareto data scaling (mean-centering and division by the square root of standard deviation of each variable) were subjected to principal component analysis (PCA) for examination of the intrinsic variation in the NMR data set and similarities in variables. Subsequently, Partial Least Squares Discriminant Analysis (PLS-DA) was performed to maximize the class discrimination using the MetaboAnalyst 5.0 program (https://www.metaboanalyst.ca) equipped with functions of the R software^57^. Score plots and heatmaps of the urinary metabolite data were also generated to enumerate the variations in pregnant and non-pregnant animals at different days of introspection. Further, Orthogonal Partial Least Squares Discriminant Analysis (OPLS-DA) and enumeration of Variable Importance in Projection (VIP) score of the metabolites contributing to the group difference between the pregnant and non-pregnant samples was also performed. Receiver operating characteristic–Area Under Curve (ROC-AUC) analysis was carried out employing MetaboAnalyst software to analyze the cut-off values of the metabolites diagnostically relevant to determine the pregnancy or open status of the animals at different days of the experiment. Pathways related to the profoundly varied metabolites were also subsequently analysed.

### Metabolic pathway analysis

Metabolic pathways related to the profoundly varied metabolites were subsequently analysed through MetaboAnalyst 5.0. The Pathway Analysis program employed a combination of pathway enrichment analysis and pathway topology analysis to precisely identify the most relevant metabolic pathways involved in the establishment and progression of healthy pregnancy. The KEGG-metabolic pathway library of *Bos taurus* was used to elucidate the course of pregnancy-associated metabolite dynamicity.

## Supplementary Information

Supplementary Information 1.

Supplementary Information 2.

Supplementary Information 3.

Supplementary Information 4.

Supplementary Information 5.

Supplementary Information 6.

Supplementary Information 7.
