# Supplementary figures and images for: Exploration of urinary metabolite dynamicity for early detection of pregnancy in water buffaloes
Source: Sci Rep. 2022 Sep 29;12:16295. doi: 10.1038/s41598-022-20298-1 (PMC9523026; doi:10.1038/s41598-022-20298-1)

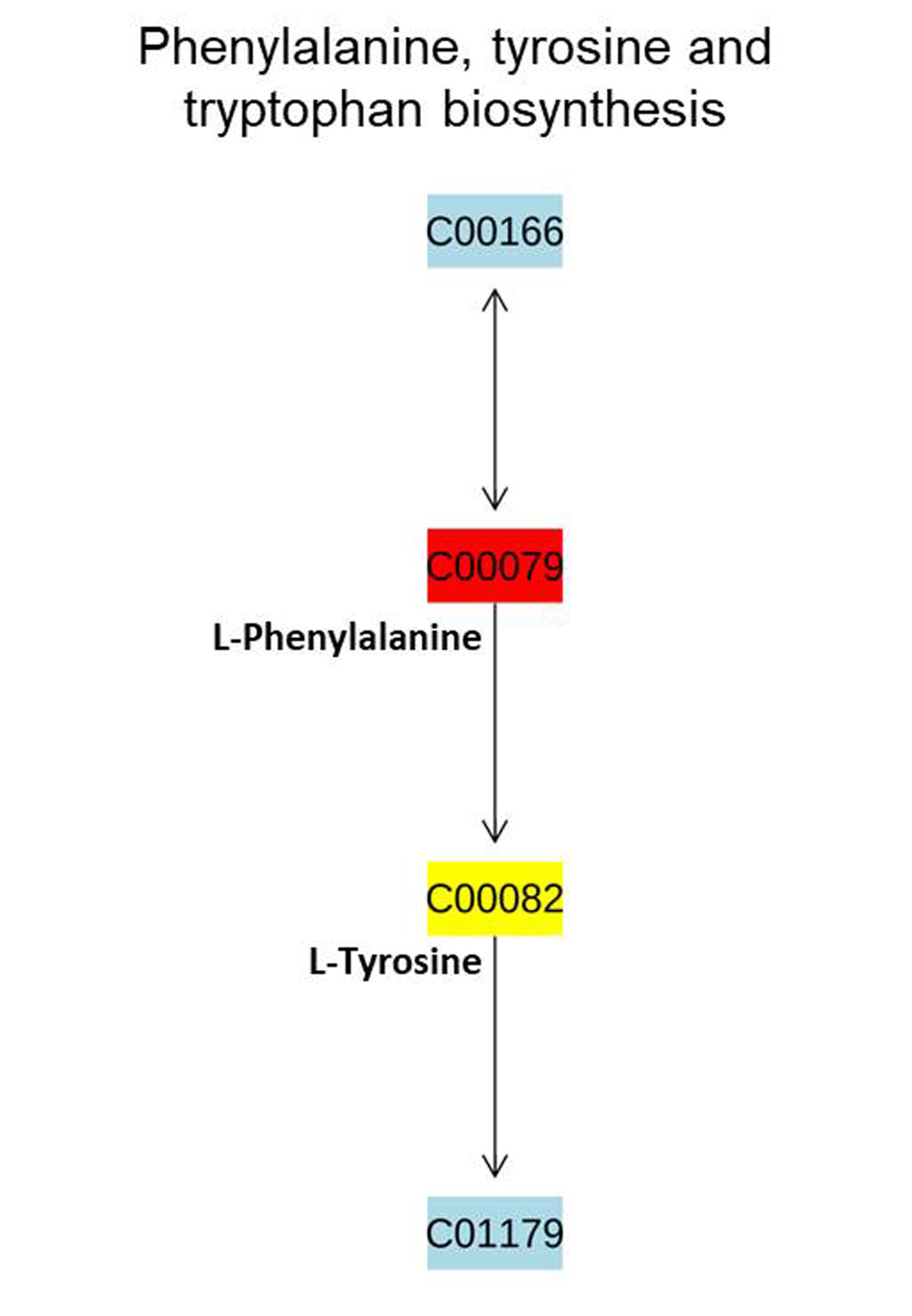

Supplement: Supplementary file 1 — Supplementary Information 1. [file 41598_2022_20298_MOESM1_ESM.jpg]

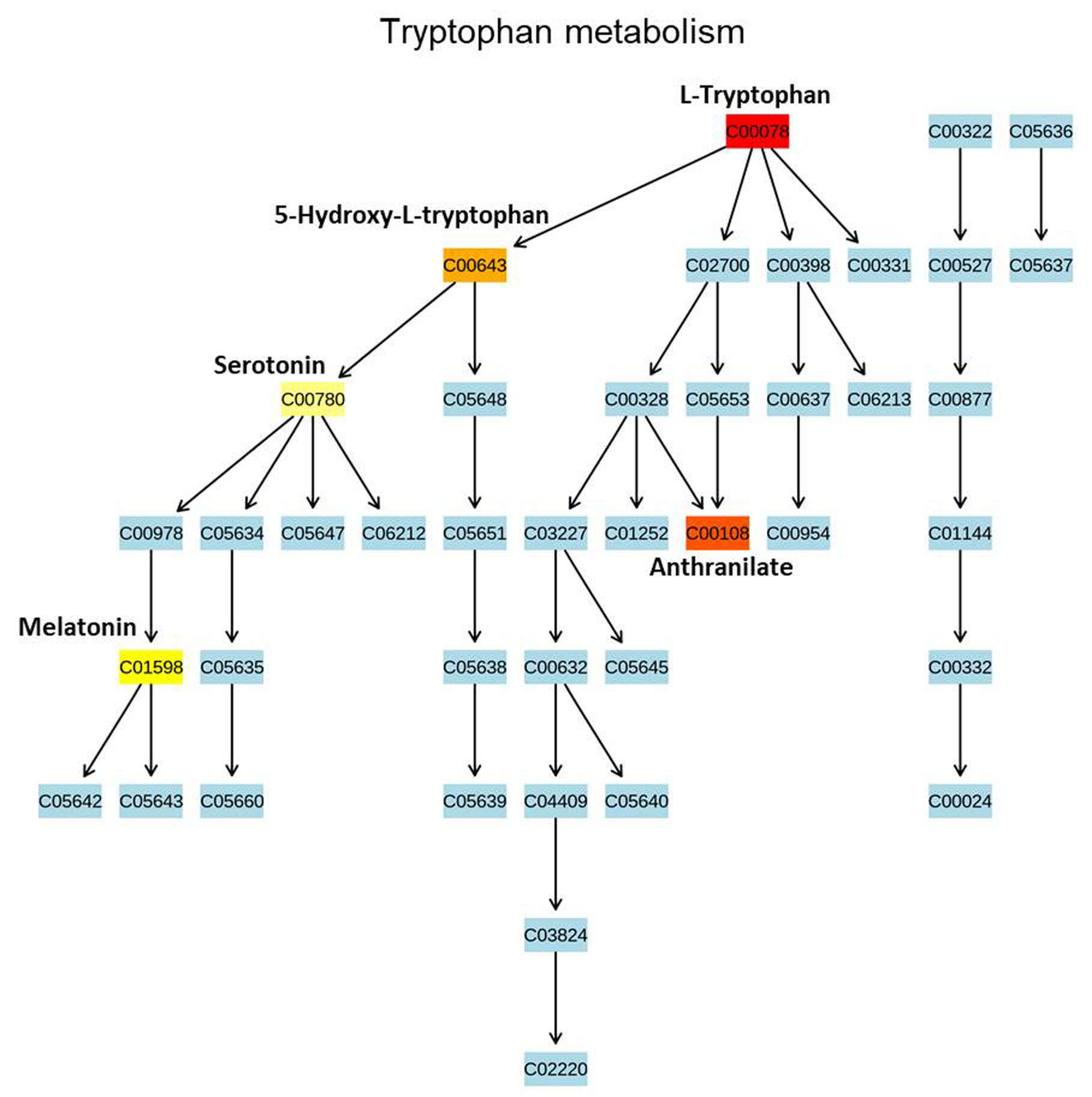

Supplement: Supplementary file 2 — Supplementary Information 2. [file 41598_2022_20298_MOESM2_ESM.jpg]

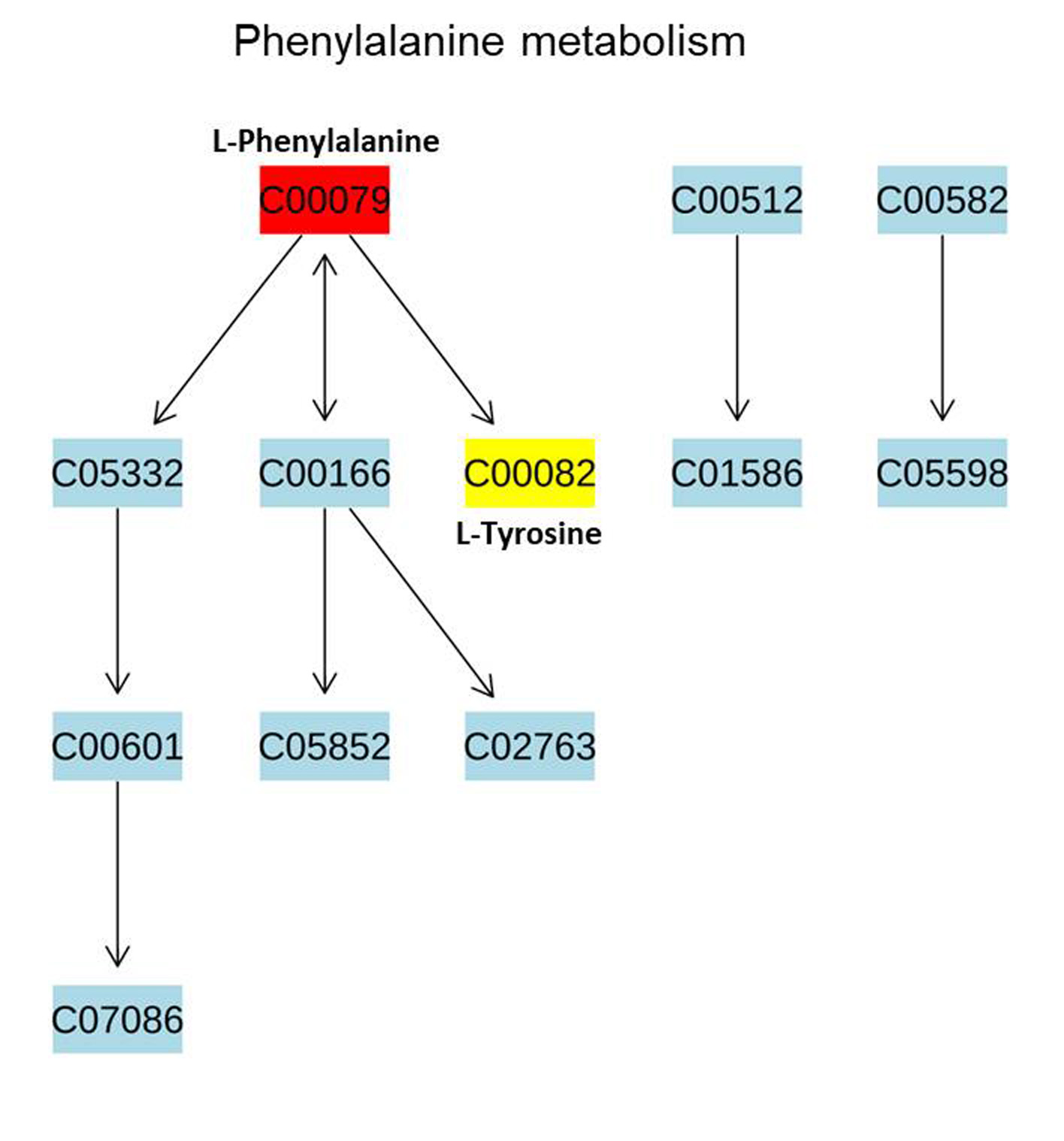

Supplement: Supplementary file 3 — Supplementary Information 3. [file 41598_2022_20298_MOESM3_ESM.jpg]

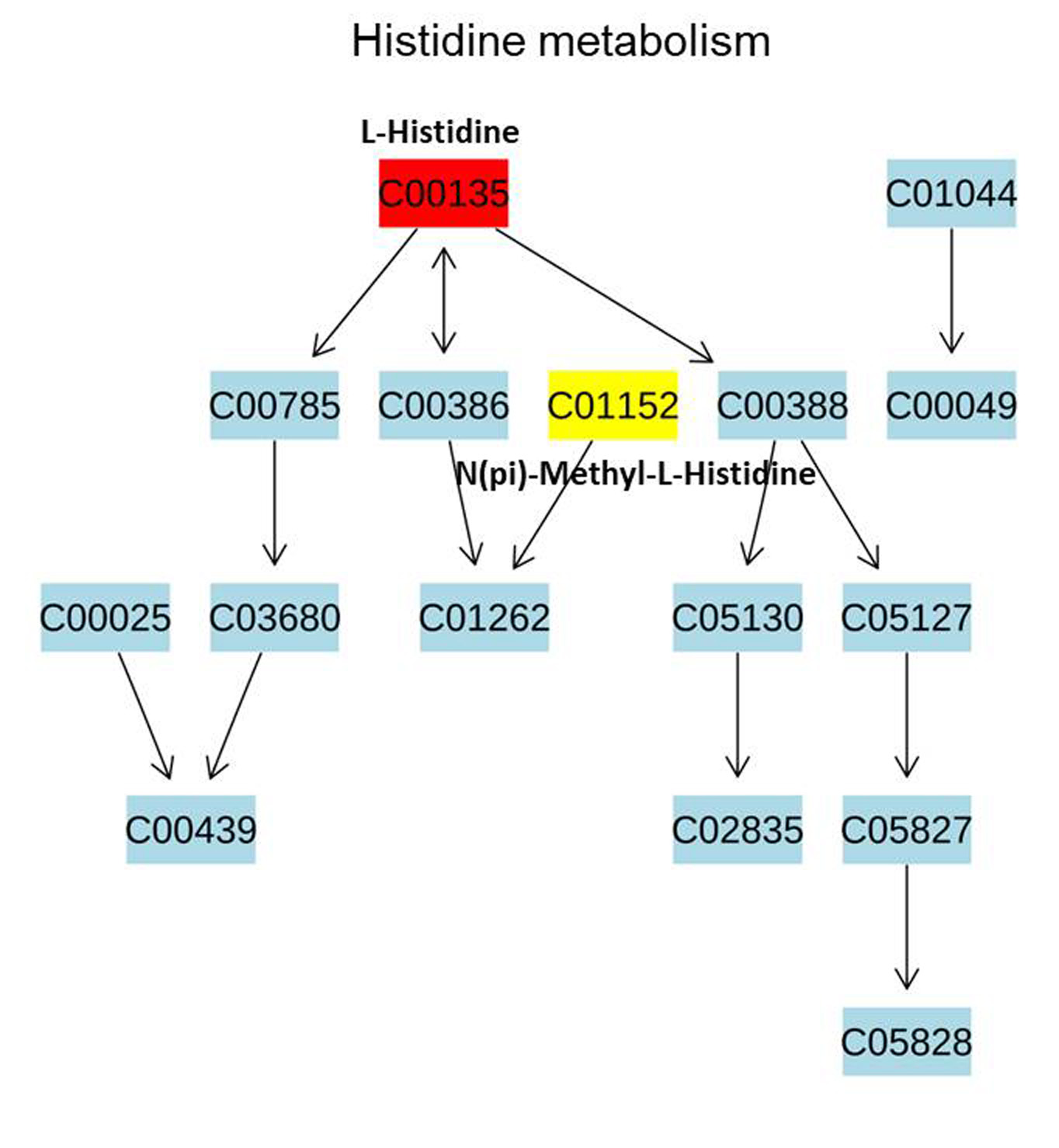

Supplement: Supplementary file 4 — Supplementary Information 4. [file 41598_2022_20298_MOESM4_ESM.jpg]

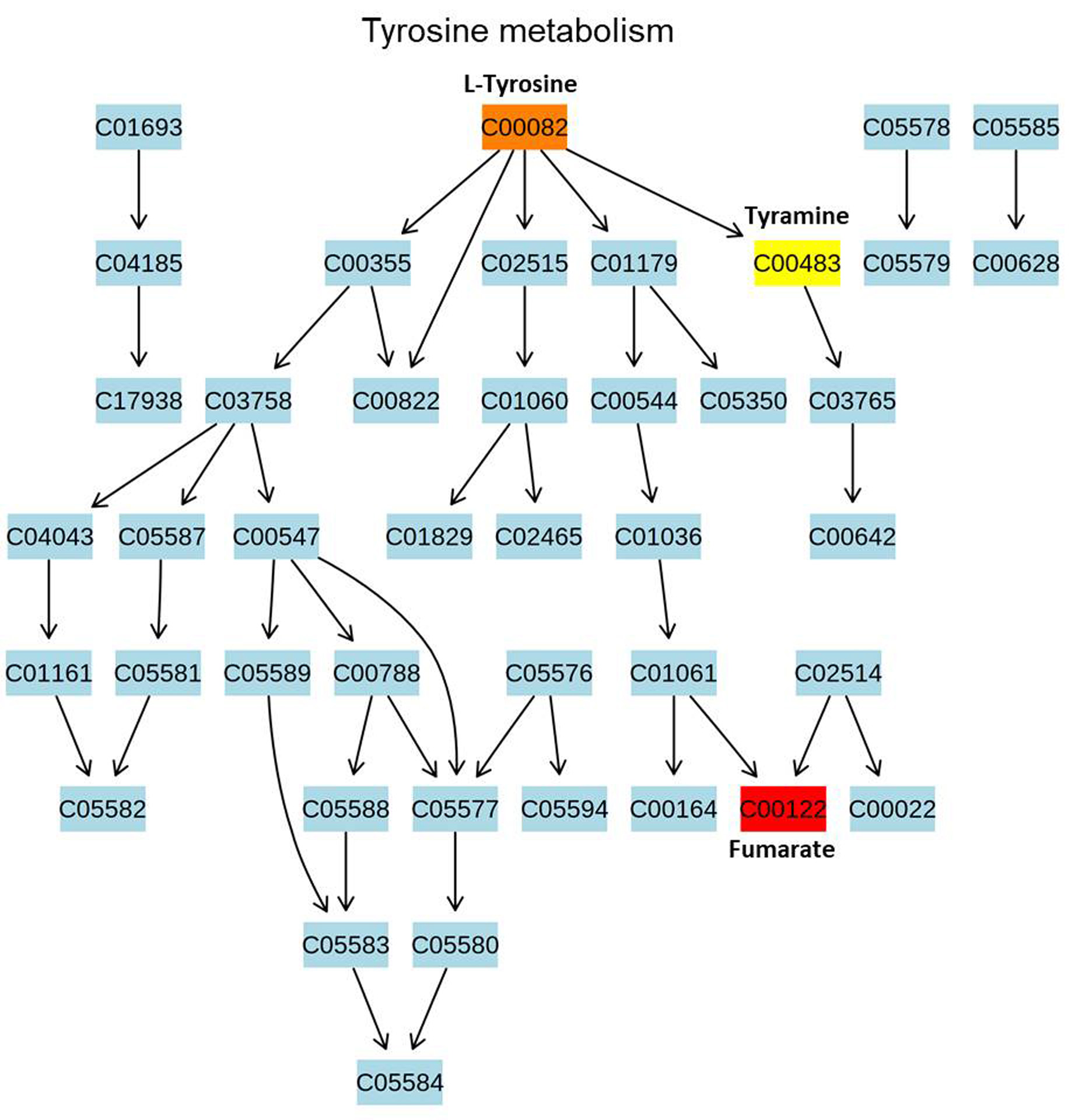

Supplement: Supplementary file 5 — Supplementary Information 5. [file 41598_2022_20298_MOESM5_ESM.jpg]

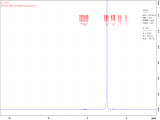

Supplement: Supplementary file 6 — Supplementary Information 6. [file 41598_2022_20298_MOESM6_ESM.zip › NMR PHD RAW DATA/0th day/NP/4924/pdata/1/thumb.png]

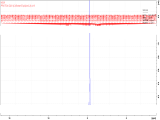

Supplement: Supplementary file 6 — Supplementary Information 6. [file 41598_2022_20298_MOESM6_ESM.zip › NMR PHD RAW DATA/10th day/NP/4539/pdata/1/thumb.png]
