# Supplementary material for: Exploration of urinary metabolite dynamicity for early detection of pregnancy in water buffaloes
Source: Sci Rep. 2022 Sep 29;12:16295. doi: 10.1038/s41598-022-20298-1 (PMC9523026; doi:10.1038/s41598-022-20298-1)
Supplement: Supplementary file 7 — Supplementary Information 7. [file 41598_2022_20298_MOESM7_ESM.docx]

**Supplementary Documents**

**Supplementary Figure S1:** Pathway and metabolites of Phenylalanine, tyrosine and tryptophan biosynthesis

**Supplementary Figure S2:** Pathway and metabolites of Tryptophan metabolism

**Supplementary Figure S3:** Pathway and metabolites of Phenylalanine metabolism

**Supplementary Figure S4:** Pathway and metabolites of Histidine metabolism

**Supplementary Figure S5:** Pathway and metabolites of Tyrosine metabolism

**Supplementary Data**

**NMR Raw Data:** Provided as zip file that can be extracted and accessed through MestReNova software 6.0.2-5475 and Chenomx NMR Suite 8.40 program (Chenomx Inc, Edmonton, Canada) to visualize the NMR peaks
