# Supplementary material for: Exploration of urinary metabolite dynamicity for early detection of pregnancy in water buffaloes
Source: Sci Rep. 2022 Sep 29;12:16295. doi: 10.1038/s41598-022-20298-1 (PMC9523026; doi:10.1038/s41598-022-20298-1)
Supplement: Supplementary file 6 — Supplementary Information 6. [file 41598_2022_20298_MOESM6_ESM.zip › NMR PHD RAW DATA/18th day/Pregnant/P 18.pdf]

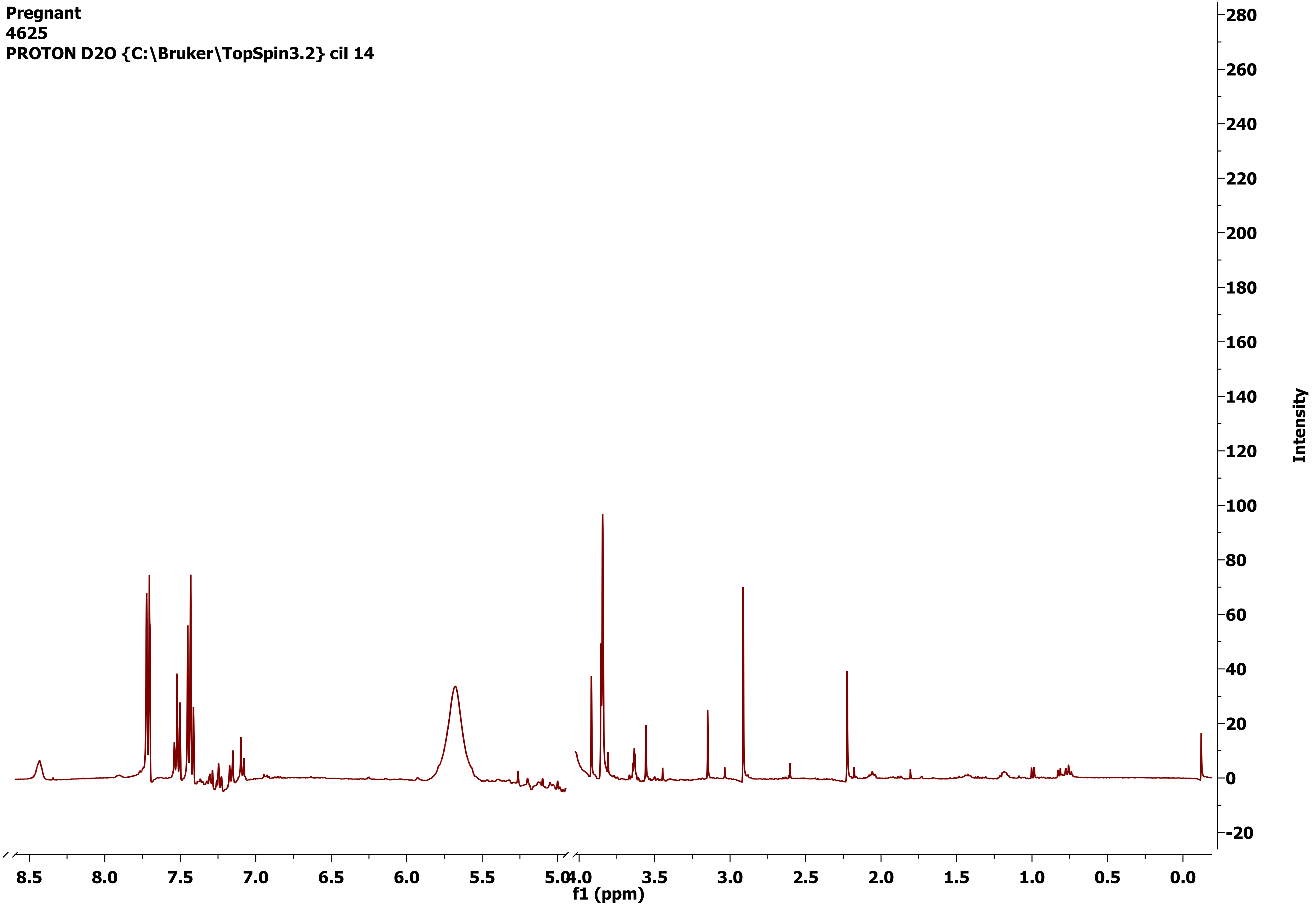

Pregnant
4625
PROTON D2O {C:\Bruker\TopSpin3.2} cil 14
Intensity
280
260
240
220
200
180
160
140
120
100
80
60
40
20
0
-20
8.5
8.0
7.5
7.0
6.5
6.0
5.5
5.0
4.0
3.5
3.0
2.5
2.0
1.5
1.0
0.5
0.0
f1 (ppm)
